# Rapid Detection of *Vibrio vulnificus* with Recombinase Polymerase Amplification Assays Based on Specific Sequence Tags of Core Genome

**DOI:** 10.3390/microorganisms14020496

**Published:** 2026-02-19

**Authors:** Bing Yuan, Jianhao Xu, Jiaxin Zhang, Jinglin Wang, Yuan Yuan

**Affiliations:** State Key Laboratory of Pathogen and Biosecurity, Academy of Military Medical Sciences (AMMS), Beijing 100071, China; yuanbing3008@126.com (B.Y.); xu1996qinqin@163.com (J.X.); zjx349793026@163.com (J.Z.)

**Keywords:** *Vibrio vulnificus*, RPA assay, core genome, specific sequence tag, rapid detection, seafood

## Abstract

*Vibrio vulnificus* (*V. vulnificus*) is a motile, Gram-negative, opportunistic human pathogen capable of causing severe to life-threatening infections in individuals with predisposing conditions. It has the highest mortality rate among foodborne pathogens. Rapid and accurate detection of *V. vulnificus* is crucial for preventing and controlling acute deaths caused by infection with this bacterium. However, identifying *V. vulnificus* is challenging due to its high genomic plasticity. We analyzed 518 *V. vulnificus* genomes to construct large-scale pan-genomes and selected specific sequence tags in their core genomes that effectively distinguish *V. vulnificus* from its closely related species. Specifically, one specific sequence tag with the minimal mutations was selected for *V. vulnificus* detection, combined with a recombinase polymerase amplification (RPA) method. The results showed that the developed RPA detection method displayed high specificity and enabled the identification of a specific 462 bp band from *V. vulnificus*. The reaction involved isothermal incubation at 39 °C for 20 min with a compact portable instrument. The sensitivity of the method was determined to be as low as 0.5 aM (1.65 fg/μL) of genomic DNA, 0.96 copies/μL of pUC57-Vv plasmid and 1 CFU/mL of *V. vulnificus* cells. The RPA method accurately detected target DNA within 5–10 min. Additionally, specificity testing was performed using 33 different strains of *V. vulnificus*. In conclusion, the established RPA method exhibits excellent high sensitivity and rapid discriminative capability, making it suitable for clinical applications and rapid detection in the field.

## 1. Introduction

*V. vulnificus* is a Gram-negative bacterium widely distributed in seawater and aquatic products, and most illnesses resulting from vibrio infection involve *V. vulnificus*, *Vibrio cholerae* and *Vibrio parahaemolyticus*. These bacteria predominantly cause clinical gastroenteritis, wound infections, and septicemia. Infections typically arise following consumption of raw seafood or exposure to contaminated seawater or freshwater. Currently, most coastal countries in the world have reports of pathogenic *V. vulnificus* [1,2]. *V. vulnificus* inhabits the marine environment and must adapt to various environmental pressures and nutritional conditions, which may result in point mutations occurring in its genome [3,4]. The genomic structure of *V. vulnificus* is intricate, encompassing multiple plasmids and genomic islands that can induce genome instability, leading to the occurrence of point mutations [3,4,5]. The frequency of SNP mutations in *V. vulnificus* is high—229,462 SNP mutations were found by mutation detection in the whole genome sequences of 260 strains of *V. vulnificus* [6]. The high-frequency genomic mutations of *V. vulnificus* pose challenges to its molecular detection.

Currently, hospitals commonly use enzyme-linked immunosorbent assays (ELISA) and immunofluorescence assays to diagnose and monitor immune responses to *V. vulnificus* infection. However, these assays are time-consuming, requiring 4 h or more for completion. Molecular techniques can complement serological assays for the diagnosis of *V. vulnificus* infection. Real-time polymerase chain reaction (RT-PCR) is highly sensitive and specific. Unfortunately, because this technique requires highly specific equipment and reagents, the utilization of RT-PCR is restricted to referral laboratories with sufficient funding and advanced equipment.

In recent years, extensive efforts have been dedicated to the development of a time-saving, simple nucleic acid-based assay that relies on portable equipment for use in resource-limited settings for the detection of various infectious diseases [7]. The recombinase polymerase amplification (RPA) method, developed by Olaf Piepenburg [8], can be used to amplify the target gene under isothermal temperature conditions within 5–20 min. The reaction system includes a recombinase (UvsX), a single-stranded DNA-binding protein, and a DNA polymerase. This method is fast, easy to use, and sensitive, making it suitable for clinical applications and rapid detection in the field [9,10,11,12,13,14].

With the rapid development of high-throughput sequencing technology, a large number of *V. vulnificus*-related genomic data can be obtained in a short period of time. These massive data make it possible to obtain the specific sequence tags of the core genome of *V. vulnificus* by bioinformatics analysis. In the present work, we developed an RPA assay to detect the specific sequence tags of the core genome of *V. vulnificus* using a compact portable Genie-II instrument (OptiGene, Horsham, UK). The Genie-II is equipped with a lithium battery and can keep working for 12 h in the field. The analytical specificity and sensitivity of the assay were evaluated, with detection results confirmed by RT-PCR. To further assess the specificity of sequence tags, 33 *V. vulnificus* strains were analyzed.

## 2. Materials and Methods

### 2.1. Materials and Reagents

All reactions involved primers; probes were synthesized by Sangon Biotech (Shanghai, China). The TwistAmp^TM^ exo Kit for RPA was purchased from TwistDX (Cambridge, UK). DNase/RNase-Free distilled, deionized water (DDH_2_O) and Quantitative Realtime PCR (qPCR) Master Mix were purchased from TIANGEN Biotech (Beijing, China). The QIAamp^TM^ DNA Mini Kit, which was used to extract genomic DNA, was purchased from Qiagen (Hilden, Germany). The TIANprep Mini Plasmid Kit, which was used to extract plasmid, was purchased from TIANGEN Biotech (Beijing, China). The pUC57 recombinant plasmids containing a 462 bp specific fragment of *V. vulnificus* were transformed by E. coli, and the above process was performed by Sangon Biotech (Shanghai, China).

### 2.2. Bacterial Strains and Clinical Samples

A total of 518 *V. vulnificus* genomes were used for bioinformatics analysis in this work. The publicly available genomes were downloaded from the National Center for Biotechnology Information (NCBI) GenBank database (https://www.ncbi.nlm.nih.gov/genome/browse#!/prokaryotes/476/, accessed on 17 January 2026).

The genomic DNA of *V. vulnificus*, *Burkholderia pseudomallei*, *Burkholderia mallei*, *Brucella melitensis*, *Brucella abortus*, *Francisella tularensis*, *Bacillus anthracis*, *Yersinia pestis*, *Vibrio cholerae*, *Staphylococcus aureus*, *Vibrio parahaemolyticus*, *Salmonella typhimurium*, *Escherichia coli*, *Bacillus cereus*, *Bacillus thuringiensis*, and *Bacillus subtilis* provided by the Academy of Military Medical Sciences were used for the specificity tests.

**Figure 1:**
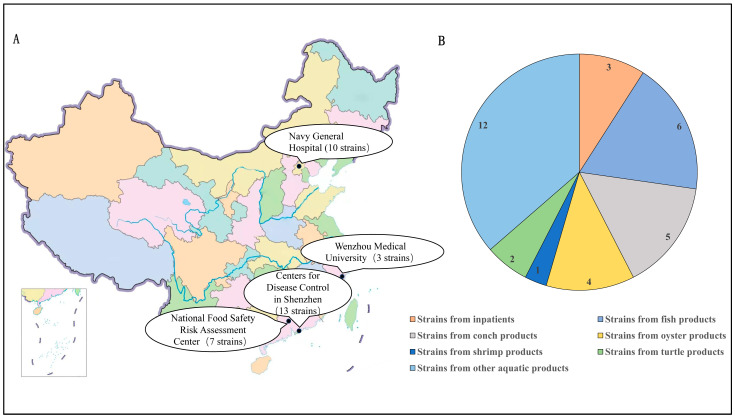
(**A**) Geographic map of thirty-three strains from Shenzhen, Beijing, Guangzhou and Wenzhou in China. Note: map lines delineate study areas and do not necessarily depict accepted national boundaries. (**B**) The proportion and number of *V. vulnificus* strains from inpatients or aquatic products.

Thirty-three *V. vulnificus* strains isolated from inpatients or aquatic products from Shenzhen, Beijing, Guangzhou, and Wenzhou in China were tested for specificity. The details of the strains are listed in Table 1 and Figure 1.

### 2.3. DNA Extraction

Total genomic DNA was extracted from samples with the QIAamp DNA Mini Kit (Qiagen, Hilden, Germany) in accordance with the manufacturer’s protocol. The purified DNA products were dissolved in 100 µL of nuclease-free water and preserved at −40 °C until use. Plasmid standards were serially diluted 10-fold, ranging from 10^9^ copies/µL to 960 copies/µL. The plasmid copy number was calculated using the following equation: DNA copy number (copies/µL) = [6.02 × 10^23^ × plasmid concentration (ng/µL) × 10^−9^]/[DNA length (nt) × 660]. The diluted plasmid standards were also stored at −40 °C until further analysis.

### 2.4. Sequencing and Assembly

Whole genome de novo sequencing was conducted on *V. vulnificus* strains using the Illumina MiSeq platform (Illumina, San Diego, CA, USA) for the construction of multiplexed paired-end libraries, which had an average insert size of 300 bp. Next, the raw short-read sequences generated from each strain through sequencing were subjected to low-quality data filtering. This filtering process was carried out using the FASTQ Quality Filter module integrated in the FASTX-Toolkit software (version 0.0.13) [15] (http://hannonlab.cshl.edu/fastx_toolkit/, accessed on 17 January 2026). Following quality control, the filtered high-quality reads were assembled with Shovill software (version 1.0.4), which is based on SPAdes 3.0. All assembly procedures were performed with the default parameter settings [16] (https://github.com/tseemann/shovill/, accessed on 17 January 2026).

### 2.5. Phylogenetic Analysis

The SNPs were identified through pairwise comparisons of 11 previously published *Vibrio* genomes (*Vibrio campbellii* DS40M4 1088888.3, *Vibrio campbellii* ATCC BAA-1116 338187.36, *Vibrio harveyi* 1DA3 673519.3, *Vibrio rotiferianus* DAT722 987060.4, *Vibrio* sp. AND4 314289.8, *Vibrio alginolyticus* strain LMG 11650 663.84, *Vibrio alginolyticus* NBRC 15630 = ATCC 17749 1219076.4, *Vibrio parahaemolyticus* V-223/04 1238219.3, *Vibrio vulnificus* YJ016 672.1601, *Vibrio vulnificus* YJ016 196600.6, and *Vibrio vulnificus* CMCP6 216895.17) using MUMmer 3.0 [17] (http://mummer.sourceforge.net/, accessed on 17 January 2026). Then, SNPs in repeated regions with low-quality scores (<20) or supported by few reads (<10 paired-end reads) were eliminated. A maximum likelihood tree (MLTree) was built using RaxML 8.0 [18] (https://cme.h-its.org/exelixis//web/software/raxml/, accessed on 17 January 2026) based on the concatenated SNPs.

### 2.6. Screening for V. vulnificus Core Genome Sequences

A total of 518 *V. vulnificus* genome sequences were annotated using Prokka [19] (https://github.com/tseemann/prokka/, accessed on 17 January 2026), a dedicated tool for rapid and standardized annotation of prokaryotic genomes with default parameter settings applied. The high-quality GFF3 format annotation files generated from Prokka were then utilized as input datasets in Roary analysis [20] (https://sanger-pathogens.github.io/Roary/, accessed on 17 January 2026). The analysis was used to conduct a comprehensive pan-genome characterization and identify the presence and absence of genes across all tested *V. vulnificus* genomes.

### 2.7. Constructing Specific Sequence Tags for V. vulnificus Based on the Core Genome Sequences

To validate the specificity of the designed sequence tags, core genome sequences of *V. vulnificus* were subjected to further sequence alignment analysis using both the local NCBI BLASTN software and the online NCBI BLAST platform (https://blast.ncbi.nlm.nih.gov/Blast.cgi/, accessed on 17 January 2026). Because of the high frequency of SNP mutations in *V. vulnificus*, complete genome sequences of multiple strains were downloaded from the NCBI database, and sequence alignment was performed between these genomic sequences and the designed *V. vulnificus* sequence tags using CLC Sequence Viewer 6 software. The identified sequence tags were confirmed to have specific alignment only with the genome sequences of *V. vulnificus.* On the basis of these comparative analyses, one specific sequence tag was selected for further experiments.

### 2.8. Design and Screening of RPA Primers and RPA Probes

The specific sequence tag was used to design the primers and the probe by Primer Premier 6 according to the principles relating to RPA primer and probe design (primer size between 30 and 35 bp, probe size between 46 and 52 bp, product size between 100 and 300 bp). The efficiency of different sets of RPA primers was tested with *V. vulnificus* genomic DNA, and the signal was examined using the Genie-II instrument (OptiGene, Horsham, UK). The RPA probe was designed using the amplified sequence of RPA primers. The 30th base of the probe was labeled with 6-carboxyfluorescein (6-FAM), the 33rd base of the probes was labeled with BHQ-1, and a base analog tetrahydrofuran (THF) was inserted to replace the 31st base, with the 3′ end blocked with C3-spacer. Oligonucleotide details are listed in Table 2.

### 2.9. Analytical Sensitivity of Real-Time RPA Assay

The sensitivity of the established real-time RPA assay was evaluated using serially diluted genomic DNA of *V. vulnificus* as a template. And the concentrations were seted at 100 aM, 10 aM, 1 aM and 0.5 aM. Each sample was analyzed in duplicate with five independent experimental runs. The assays were performed at 39 °C for 20 min using the Genie-II instrument, strictly following the manufacturer’s instructions for the TwistAmp^TM^ exo kit. The RPA reaction system was composed of 50 μL reaction solution: 29.2 μL of Primer Free Rehydration buffer, 2.1 µL of forward primer (10 µM), 2.1 µL of reverse primer (10 µM), 0.6 µL of probe (10 µM), 3 µL of MgOAc, and 13 µL of template DNA, bringing the total volume to 50 μL.

For further sensitivity validation, a recombinant plasmid pUC57-Vv containing the target *V. vulnificus* genomic fragment was constructed and used as the template. The plasmid was 10-fold serially diluted to concentrations ranging from 960 copies/μL to 0.96 copies/μL, plus an additional concentration of 0.2 copies/μL. The RPA reaction system was the same as above. In addition, the extracted nucleic acids from serially diluted *V. vulnificus* (10^4^ CFU/mL to 10^0^ CFU/mL) were also used as template to assess the sensitivity of the developed assay. In all reactions, DDH_2_O was included as the no-template control (NTC).

### 2.10. Analytical Specificity of Real-Time RPA Assay

The specificity of the RPA assay was determined using a panel of genomic DNA samples, including *Burkholderia pseudomallei*, *Burkholderia mallei*, *Brucella melitensis*, *Brucella abortus*, *Francisella tularensis*, *Bacillus anthracis*, *Yersinia pestis*, *Vibrio cholerae*, *Staphylococcus aureus*, *Vibrio parahaemolyticus*, *Salmonella typhimurium*, *Escherichia coli*, *Bacillus cereus*, *Bacillus thuringiensis*, and *Bacillus subtilis.*

Also, to evaluate the specificity of the RPA assay directly on clinical samples, 33 *V. vulnificus* strains collected from inpatients or aquatic products were tested using the established RPA methods in this study. All the RPA test results were compared with those from the standard RPA assay.

### 2.11. Detection of Simulated Blood Samples Using Real-Time RPA Assay

To assess the applicability of the established RPA assay for the detection of *V. vulnificus*, a total of 14 simulated blood samples were prepared. In a double-blinded experiment, the samples were spiked with *V. vulnificus* at concentrations ranging from 10^3^ to 10^0^ CFU/mL. Meanwhile, three blank control (BC) samples were prepared using DDH_2_O.

DNA was extracted with the QIAamp DNA Mini Kit, and 13 μL of the extracted nucleic acid were used for RT-PCR and the real-time RPA assay. The RT-PCR assay for *V. vulnificus* was performed according to a previously published method [21] in the qTOWER3G instrument (Analytikjena, Jena, Germany). The thermal cycling conditions were as follows: pre-denaturation at 50 °C for 30 min and 95 °C for 10 min, followed by 40 cycles of denaturation at 95 °C for 15 s and annealing-extension at 60 °C for 60 s. Finally, the detection results obtained from RT-PCR and RPA assays were systematically analyzed and compared.

## 3. Results

### 3.1. Acquisition of V. vulnificus Core Genome Sequences

**Figure 2:**
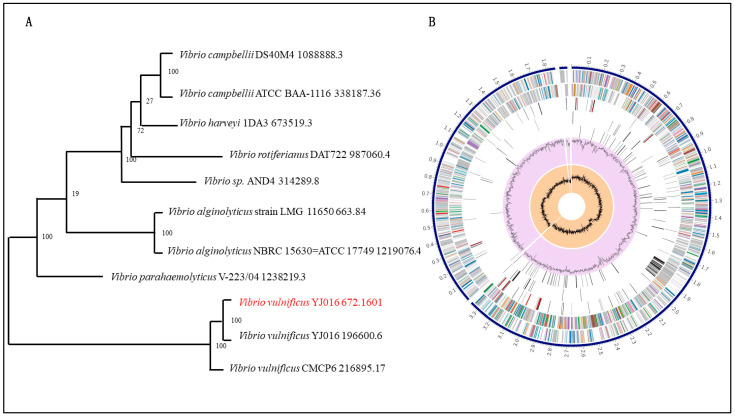
(**A**) Evolutionary relationships among the genus *Vibrio*, including 11 genomes of eight species. The rooted tree was constructed using maximum likelihood. (**B**) The genome annotation of *V. vulnificus* YJ016. This includes, from outer to inner rings, the distribution of the *V. vulnificus* tags, the contigs, CDS on the forward strand, CDS on the reverse strand, RNA genes, CDS with homology to known antimicrobial resistance genes, CDS with homology to known virulence factors, GC content, and GC skew.

A total of 518 *V. vulnificus* strains were selected for whole genome analysis in this study. Phylogenetic trees were constructed to exhibit the genetic distances and inter-individual relationships within the population, which provides an effective approach for the research on population structure and species evolution (Figure 2A). The genome annotation of *V. vulnificus* YJ016 was performed using Prokka software, with a GFF3 file (Figure 2B). After the pan-genome sequence of *V. vulnificus* was identified using Roary software, Python programming was used to extract the core genome sequence from the obtained pan-genome.

### 3.2. Obtaining V. vulnificus-Specific Sequence Tags

**Figure 3:**
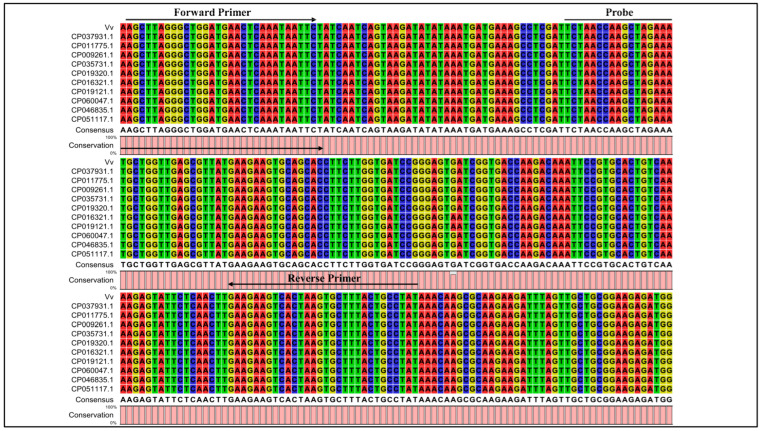
The partial sequences of the *V. vulnificus*-specific sequence tag (Vv) in this study were evaluated by sequence alignment with ten *V. vulnificus* strains (primer and probe regions listed).

Sequences fragments on the *V. vulnificus* core genome with a length of >5000 bp were chosen to boost detection specificity. In total, nine *V. vulnificus*-specific sequence tags were screened using a local version of the NCBI BLASTN and online NCBI BLAST that contains all the public genomes to date (Supplementary Material). The results showed that all nine *V. vulnificus*-specific sequence tags were identical to the sequences of the *V. vulnificus* species. Also, the *V. vulnificus*-specific sequence tags were inconsistent with non-*V. vulnificus* species and strains and could therefore be used to identify *V. vulnificus*. In addition, the complete genome sequences of several *V. vulnificus* strains were downloaded from NCBI and compared with the sequences of *V. vulnificus* sequence tags using CLC Sequence Viewer 6 software. As shown in Figure 3, 10 strains of *V. vulnificus* were compared with the sequences of *V. vulnificus* sequence tags. Finally, one *V. vulnificus*-specific sequence tag was selected to detect *V. vulnificus* and named Vv (Figure 3).

### 3.3. Optimal RPA Primers

**Figure 4:**
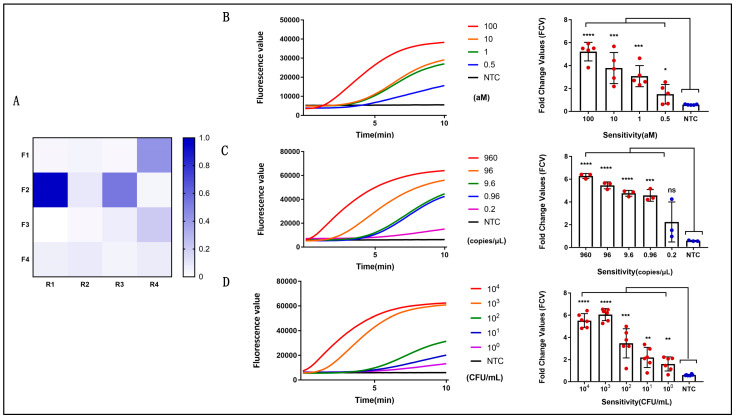
RPA assay for *V. vulnificus*. (**A**) Screening of optimal RPA primers under the same DNA template gradient concentration. (**B**) Sensitivity evaluation using genomic DNA of *V. vulnificus*, which can reach 0.5 aM, **** (*p* < 0.0001), *** (*p* < 0.001), * (*p* = 0.0430), ns (non-significant), *n* = 5 technical replicates. (**C**) Sensitivity evaluation by pUC57-Vv plasmid, which can reach 0.96 copies/μL, **** (*p* < 0.0001), *** (*p* < 0.001), ns (non-significant), *n* = 3 technical replicates. (**D**) Sensitivity evaluation using *V. vulnificus*, which can reach 1 CFU/mL, **** (*p* < 0.0001), *** (*p* < 0.001), ** (*p* = 0.0033), *n* = 6 technical replicates. The fluorescent value–time curves of the RPA assay presented are from one representative experiment (the right graph of (**B**–**D**)).

The designed primers and probes matched the genome sequence of *V. vulnificus* and showed no cross-reactivity with sequences from non-*V. vulnificus* strains. In total, four forward primers and four reverse primers were generated, and 16 combinations of candidate primer pairs were prepared and screened for reactivity with genomic DNA. Of these primer sets, primer set Vv-F2/R1 showed the highest sensitivity with the probe, as reflected by the maximum fluorescence value. Consequently, the primer set composed of Vv-F2 and Vv-R1 was selected and used as the primer set for the following detection (Figure 4A).

### 3.4. Evaluation of the Sensitivity of the RPA Assay

The sensitivity of the RPA assay for *V. vulnificus* detection was determined using a panel of serially diluted bacterial genomic DNA, recombinant plasmids harboring the specific sequence tag Vv, and *V. vulnificus* cells. As shown in Figure 4B–D, the fluorescence signal increased with the number of aM, copies, or CFUs, and the detection time of the positive reaction was 5–10 min. To validate the stability of the sensitivity, the experiment was repeated three to five times. The results showed that the sensitivity of the method was determined to be as low as 0.5 aM (1.65 fg/μL) of genomic DNA (Figure 4B), 0.96 copies/μL of pUC57-Vv plasmid (Figure 4C) and 1 CFU/mL of *V. vulnificus* cells (Figure 4D), indicating that this method was ultrasensitive.

### 3.5. Evaluation of the Specificity of the RPA Assay

**Figure 5:**
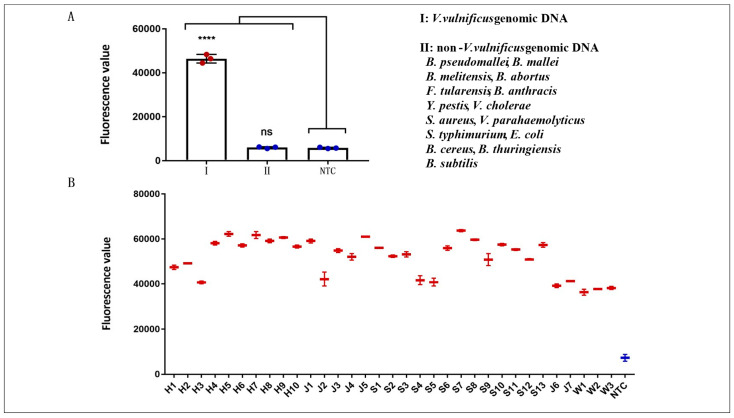
Specificity evaluation of RPA assay. (**A**) Specificity evaluation of RPA assay with 15 non-*V. vulnificus* genomic DNAs. **** (*p* < 0.0001), ns (non-significant), *n* = 3 technical replicates. (**B**) Specificity evaluation of 33 *V. vulnificus* strains from inpatients or aquatic products from Shenzhen, Beijing, Guangzhou, and Wenzhou in China. Data are presented as mean ± standard deviation (SD) of two independent replicates.

The specificity of the assays in this study was evaluated with the genomic DNA of various pathogenic bacteria in our laboratory. The non-*V. vulnificus* genomic DNA was composed of 15 bacterial genomic DNA, including *B. pseudomallei*, *B. mallei*, *B. melitensis*, *B. abortus*, *F. tularensis*, *B. anthracis*, *Y. pestis*, *V. cholerae*, *S. aureus*, *V. parahaemolyticus*, *S. typhimurium*, *E. coli*, *B. cereus*, *B. thuringiensis*, and *B. subtilis*. *V. vulnificus* genomic DNA (33 fg/µL) was 100 times lower than that of the non-*V. vulnificus* genomic DNA (3.3 pg/µL). The results of the RPA assay are shown in Figure 5A. In contrast with the other bacterial samples and water control, only *V. vulnificus* samples produced amplification signals. Hence, the RPA assay for the detection of *V. vulnificus* was 100% specific. Furthermore, the results of the RPA assays for *V. vulnificus* strains isolated from inpatients or aquatic products from Shenzhen, Beijing, Guangzhou, and Wenzhou in China were shown in Figure 5B. All 33 samples produced amplification signals. Hence, the RPA assay can detect all positive samples and has excellent specificity.

### 3.6. Evaluation of the RPA Assay Using Simulated Blood Samples

**Figure 6:**
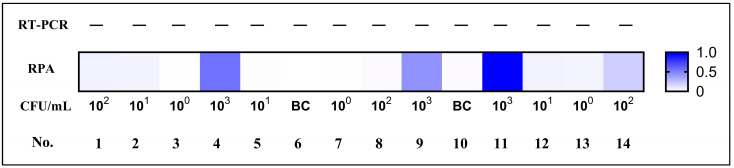
Simulated blood samples were analyzed using both the RPA and RT-PCR assays.

After completion of the sensitivity and specificity evaluations, the feasibility of the assay for clinical adaptation was analyzed using simulated blood samples. Human blood was added to the simulated samples. Concomitantly, RT-PCR was used as an auxiliary reference experiment. The feasibility of the RT-PCR assay was analyzed, and it was used to quantify the *V. vulnificus* spike in the simulated samples. As shown in Figure 6, *V. vulnificus* was detected in the simulated blood samples by the real-time fluorescence RPA method at 10^3^ CFU/mL, and there was no positive signal by RT-PCR. There was no cross-reaction with the human blood genome (no positive signal in the blank control group), and the result accuracy was as high as 100%, indicating that the *V. vulnificus* RPA detection technique established in this study had good sensitivity and specificity.

## 4. Discussion

*V. vulnificus* is widely found in marine and estuarine environments and can cause illness in humans and fish, leading to wound infections and even sepsis [22]. It is commonly found in men and the elderly with underlying diseases, such as those with hepatitis, cirrhosis, diabetes, and low immunity [23]. *V. vulnificus*, *V. cholerae* and *V. parahaemolyticus* are collectively known as the three major pathogenic vibrions. Currently, the latter two vibrions have caused global epidemics [24,25]. *V. vulnificus* has not yet caused a pandemic or outbreak, but the number of infections caused by it is increasing, and it has the highest mortality rate among foodborne pathogens. A study in the United States has shown that the number of patients associated with *V. vulnificus* has been increasing in recent years, and *V. vulnificus* in seafood-related foodborne pathogens can account for up to 95% of mortalities [26]. Therefore, a more rapid and sensitive detection method is required for monitoring *V. vulnificus* infection and for preventing and treating diseases caused by this pathogen.

Various methods have previously been attempted to identify *V. vulnificus*. However, due to the high SNP mutation frequency in *V. vulnificus*, it is difficult to detect and identify. To remedy these deficiencies, nine specific sequence tags of *V. vulnificus* were identified by bioinformatic analysis, and one of these tags was used to develop an RPA detection method. Notably, the selection of specific targets on core genomes was more conservative and stable and had fewer SNP mutations. The other eight specific sequence tags will also provide important clues for the development of molecular diagnostic techniques for *V. vulnificus* in future studies.

The RPA primers and probe we designed were based on the selected sequences of *V. vulnificus*. Based on the selected primer pairs, sensitivity evaluation and simulation-sample evaluations were conducted. In addition, 33 *V. vulnificus* strains isolated from inpatients or fish, conch, oyster, shrimp, turtle and other aquatic products were obtained from Shenzhen, Beijing, Guangzhou, and Wenzhou in China. The RPA assays performed on the 33 *V. vulnificus* strains from different regions in China generated amplification signals, indicating that the RPA detection method used to detect *V. vulnificus* had high specificity. Hence, depending on the advantages of specificity, rapidity, and ultrasensitivity, the RPA assay developed in this study will play a significant role in the accurate and rapid diagnosis of *V. vulnificus* in clinical and field settings. In future studies, additional work such as extraction-free nucleic acid processing for clinical and environmental samples and room-temperature storage reagents for RPA could be developed to translate this method into broader application scenarios in community clinics, farmers’ markets, customs and grassroots units. Non-laboratory personnel need only take 2–5 μL of the sample lysate, add it to the reaction reagent that can be stored at room temperature, and mix well. The test result can then be visualized within 5–20 min using a portable compact instrument or a commercial lateral flow strip.

## Figures and Tables

**Table 1:** Strain information.

Strain Number	Source Number	Strain Source
S1	Vv19001	Centers for Disease Control in Shenzhen
S2	Vv19002	
S3	Vv19004	
S4	Vv19005	
S5	Vv19006	
S6	Vv19007	
S7	Vv19008	
S8	Vv19009	
S9	Vv19010	
S10	Vv19011	
S11	CS2004	
S12	CS2005	
S13	CS2006	
H1	FC1671	Navy General Hospital
H2	FC1672	
H3	FC1673	
H4	FC1679	
H5	FC1680	
H6	FC3054	
H7	FC3063	
H8	FC3066	
H9	FC3069	
H10	FC3072	
J1	18CS1	National Food Safety Risk Assessment Center
J2	18CS38	
J3	18CS58	
J4	CICC10383	
J5	CICC21615	
J6	18CS10	
J7	18CS20	
W1	QT9424	Wenzhou Medical University
W2	QT9518	
W3	QT9520	

**Table 2:** The involved oligonucleotide sequences.

Name	Sequence (5′-3′)
Vv-F1	GCTTAGGGCTGGATGAACTCAAATAATTCT
Vv-F2	AGCTTAGGGCTGGATGAACTCAAATAATTC
Vv-F3	AAGCTTAGGGCTGGATGAACTCAAATAATT
Vv-F4	CTTAGGGCTGGATGAACTCAAATAATTCTA
Vv-R1	ATAGGCAGTAAAGCACTTAGTGACTTCTTC
Vv-R2	TATAGGCAGTAAAGCACTTAGTGACTTCTT
Vv-R3	TTATAGGCAGTAAAGCACTTAGTGACTTCT
Vv-R4	TAGGCAGTAAAGCACTTAGTGACTTCTTCA
Vv-P	TCTAACCAAGCTAGAAATGCTGGTTGAGCG/i6famdt/idsp/A/ibhq1dt/GAAGAAGTGCAGCAC[C3-Spacer]
qPCR-vvp-F	TCTCGGTCTTATGCTTGTTGCA
qPCR-vvp-R	TCGGAGACGGACACCATTTC
qPCR-vvp-P	[FAM]-CATGTGTTCTCTTCCAGTCACCGCTGC-[TAMRA]

## Data Availability

The original contributions presented in this study are included in the article/Supplementary Material. Further inquiries can be directed to the corresponding authors.

## Supplementary Materials

The following supporting information can be downloaded at https://www.mdpi.com/article/10.3390/microorganisms14020496/s1, *V. vulnificus* (YJ016)-specific tags were identified by bioinformatic analysis.
