# Supplementary material for: Rapid Detection of Vibrio vulnificus with Recombinase Polymerase Amplification Assays Based on Specific Sequence Tags of Core Genome
Source: Microorganisms. 2026 Feb 19;14(2):496. doi: 10.3390/microorganisms14020496 (PMC12943105; doi:10.3390/microorganisms14020496)
Supplement: Supplementary file 1 [file microorganisms-14-00496-s001.zip › microorganisms-4147502-supplementary.pdf]

Supplementary information.

9 *V. vulnificus* (YJ016)-specific tags were identified by bioinformatic analysis.

Tag-1:>910_5343

ACATCGTATTCTTGGCGAATTTGCTGTGCGTTCACATCCACACCAACATGTTGATTCACCTTAAACTC
AACCCCTGCTTCTGCCATGAGATTGATCTTGCGGTCAATGATGTCCATGCCCAGTTTGAAATCTGGG
ATACCGAAACGCAGTAGACCACCCACTTTCTCATCGCGCTCGAAAACCGTCACCGAATGGCCTGCA
CTATTGAGCTGCTCAGCTGCCGCTAAGCCTGCCGGACCGGAACCGATGATCGCCACTTTCTTACCT
GTGCGCGAACGCGGAGTTTTGGGTTTGGCGTACCCTTCACGATACGCCGTCTCCACAATGGTTTTC
TCAATGTTACAGATGGTGATTGGGTCTTGGTTGATGCCCAATACACAAGCACTTTCACATGGCGCAG
GGCAAACTCGTCCGGTGAACTCTGGGAAGTTGTTGGTTGAACTTAGAATGTTCCACGCTTCTTCCC
AACTGTCGCGATAAACCGCATCGTTAAATTCCGGAATGATGTTACCAATTGGGCAGCCGTTGTGACA
AAACGGCACACCACAGTCCATACAACGAGACGCTTGAGTATTGATTTTCTTACCAAATTCCGCGTTG
AGTACGAACTCTTTGTTGTTTTTGATTCGCTCTGCTGGTGCAAGCTTCTTTGGAAGCTCGCGTCCAA
ACTCTAAAAATCCAGTCGGCTTACCCATTACACTGCCTCCGCTTCACGTTGCTCTGCTTCTGCCTTG
CGTTTTTGTAGTACCGCTTTGTAATCACGTGGCATGACTTTTACCAAGGATTTCAAGCTGGTTTCAAA
ATTAGCAAGGAAAGCTTGAGCCACTTCACTTCCTGTGAACTGAACATGTTTGTTCAACATATCCAGCA
GTAGATCTTTGTCTTCTTGTTCGATTGGATCGAGGTCAACCAGTTCAGGGTTCAGTTTCGACTGGAA
GTCACCTGATTTGTCCCAAACGTAGGCCACGCCGCCACTCATACCGGCGGCAAAGTTGCGACCTGT
TGAACCGAGGATAATTGCGACACCACCGGTCATGTATTCACAACCGTGGTCACCCACGCCTTCAAC
GACCACTTTGGCACCGGAGTTACGAACACAGAAACGCTCGCCAGCCAGGCCACGAATGTAAGATTC
ACCGGATGTTGCACCGTAGAAACAAACGTTACCCACGACGATGTTGTCTTCTGGCACCAGTGTTGA
GTTGCTGTCTGGGTAGAGCACCAGCGTACCGCCAGAGAGGCCTTTGCCCCAGTAGTCGTTCGCAT
CGCCTTCCACTTCAAACGTCACGCCTTTCGCGAGGAAAGCACCAAAGGATTGGCCGGCAGAACCT
TTGAACTTGACGTTCATTGGTTGTGGTAAACCTTGGTCTTTGTACACTTTCGAAATTTCGTTCGACAG
CATGGTACCCACAGAGCGATCGGTGTTGATGATTGGGAATTCCGCGGTTACCGCCAAACCGTCTTC
CAGTGCTGGTGTCGCCACTTGAATCAATTGACGATCAAGTACCGCTTCTAGCTGGTGGTTTTGTTTG
GTTTGACAGTAAATACCATCTTGCTCGCGAGGCTGCTCTAGGAAGAGAACTGGAGAAAGATCCAAAT
TCTTGTATTTCCAATGAGAAACGTTGTCGCGTACTTTGAGTTTTTGCGCCTGACCCACCATCTCATCC
ACAGTACGGAAGCCAAGTTCCGCCATGATTTCACGCACGCCTTGTGCCATGTAGAGGAAGAAGGTC
ACCACGTCTTCTACGCGGCCATCAAAGCGCTCACGTAGCGTTTTGTTCTGAGTTGCAATACCAACAG
GGCAAGTGTTTTTATGACACTTACGCATCATGATACAGCCTTCAACCACGAGGGCAGCGGTCGCGA
CACCCCATTCCTCAGCACCCAATAGCGTTGCGATTGCGATGTCACGAGGGGTTTTCATCTGGCCATC
GGCTTGAACTACTATACGGTTACGTAAACCGTTCTTCAGCAGCGTTTGGTGTGTTTCTGCAAGACCA
AGCTCCCAAGGGAGACCGGTGTGACGAATCGACGAAATCGGTGAAGCACCGGTACCACCATCGTG
ACCGGCAATCAGAACCACGTCTGCTTTTGCTTTTGCCACACCAGAAGCAATTGTCCCTACGCCAGC
TTCGGAAACCAGCTTAACGTTGACACGGCCTTTACGGTTGGCGTTTTTCAAGTCGTAGATCAGCTGA
GCCAAATCTTCGATCGAGTAGATGTCATGGTGTGGCGGTGGCGAAATCAAACCAACCCCAGGAGTG
GAGTGACGTGTTGCACCAATCCAATCGTCGACTTTGTCGCCCGGTAGCTGACCACCTTCGCCCGGT
TTTGCCCCTTGTGCCATCTTGATCTGAATTTCGTCAGAGTTAGTGAGGTAGTAAGAGGTCACACCAA
AACGGCCTGAAGCAACCTGTTTGATCGCAGAGCGCTCTGAGTCGCCATTCTCATTCGGTGAGAAGC
GAATTGGATCTTCACCACCTTCACCGGAGTTTGATTTCGCGCCGAGACGGTTCATCGCGACTGCTA
GAGTCGAGTGTGCCTCGTAAGAGATAGAGCCAAATGACATTGCGCCAGTGGCGAAACGTTTGACGA
TGCTCTCTACGGGTTCCACTTCATCGATTGGGATTGAACCCGCTGGATTTTTCACGAAATCAAGTTG

GCTACGTAGGGTAACGGCGTTGTCACCTTGGCGATCCACTTCTGCTGCGTATTTCTTGAACTCTGCG
TAGTCTTTGTTACGAGTAGAGTGTTGCAGCAGGTGAATGGTTTCAGGGTTAAACAGGTGTTTTTCAC
CACGTTGTTTCCACTGATAAACGCCGCCCACATCGAGCATTTGAATTGGGATTTCACGAGTTGGGTA
GCCCACGCGATGGCGCACTAAGACTTCTTTAGCGATGTCATCTAAGGTCAAACCTTGAATACGTGTA
ACGGTACCGGTGAAGTATTTGTCGACCACCGCTTTGCTGATACCAAGGGCTTCAAAAATTTGCGCAC
CGTGGTAGGACTGCAGCGTCGAAATACCCATTTTCGAGAAGATCTTTAATAGGCCGCCGTTGACCCC
TTTACGGTAGTTGTTGAAGTACTCTTCAACCGACACGTTGGCATCCAGCTTTTTACGACGTTGTAGAT
CAACGATCGTTTCAGTGACCAGATAAGGGTTCACCGCATTGGCGCCATAGCCCACGAGCGTGGCGA
AATGGTGTGTTTCGCGCGCATCACCCGTTTCCACTACGATGCCACATTTGGCGCGTAGCCCTTTACG
AATCAGGTGATGATGAACCGCACCCACTGCTAACATCGCTGGAATCGCTGCGTGGTTAGAGTTTACG
GCACGGTCAGTCAATAGAATGATGGAGTAACCGTCGATCACGGCATCTTCCGCGTACTGACAAATAC
GTTTGAGTGCGCGTTCCAGTTTGCCAGCGTCTTCGTTGGCTTGGAATACGATATCTAACGTTTTTGC
TTGCAAGTGTTCGTTGTCGATAGCACGCAGTTTTTCGAGCTCAGCGTTTGAGATCACTGGAGATTCA
AGTTCTACTTTGCGGCAATGAACCGGCGATTCTGCCAGCAGGTTCTGATCTTTACCGATGTAGGTAT
TGAGTGACATCACCATACGTTCACGGATCGGGTCGATCGGCGGGTTAGTCACCTGAGCAAACAGCT
GCTTGAAGTAGTTTGAAAGATGTTGCGATTGGTGTGACAAAATCGCGACTGGCCAGTCCGCACCCA
TCGAGCCAAGTGGTTCATAGCCGGTTTGTGCCAACGGCAGAATGATCTCATTCACTTCTTCTGCACT
AATGCCAAACGCTTGCTGGCGATGCAGTAGACGCTCTGGAGAAGGTTGGCTGTGTACGTTATCGGC
ATCTGGCAGCGATTTTAGGCTGAGCAGATTGTCCGTTACCCACTGCTCATACGGCTGAGCGTTGGC
AATCGAATCTTTTACTTCTTCATCGGAAATGATGCGGCCTTGTTCGAGATCGGCGACGAAGATGCGC
CCTGGCTGCAAACGGCCACGGTATTCAACGTTTTCTGGTGGGATTTCCACTACGCCAGATTCGGAT
GCCATGATCAGCATGTTGTCTTTTGTCACGGTATAGCGTGACGGGCGCAGACCGTTACGGTCTAGT
GTTGCACCGACTTGTACACCATCGGTAAAGCAGACAGACGCAGGGCCATCCCACGGTTCCATGATG
TTGGCATGGTATTGATAGAAGGCACGGCGTTTTGGATCCATGGTTTTGTTTTCTTGCCACGCTTCAG
GAATCATCATCATCAGCGCGTGCGGTAGGCTTCGGCCTGAAAGCACCAGCAGTTCCAACACCATGT
CAAAGTTGGCAGAGTCAGAAGCGCCTTCCTGGCAGATCGGCAATAGCATGTCGATCTCTGCTTGCG
TGAAGAGCTTAGATTCTAAGATCGCTTCGCGTGCTTTCATCCAGTTAAGGTTGCCGCGAACCGTGTT
GATTTCACCGTTGTGCGCAATGTAACGGAAAGGTTGAGCCAAGCGCCATTTCGGGAAAGTATTGGTA
GAGAAACGAGAGTGCACAAGAGCCAGTGCGGTGACCATGGTCGGGTTTTGTAGGTCTTGGAAATAC
TGAGGCACTTGCTCAGTGGTTAGCTGACCTTTATAGACCAAGGTTTTGTAAGACATCGAGTTGATGTA
GAAGTCATCCCCGATATTCGAAACGCTTTCAAGGCAAACGCGTACGGTGTAGTTACGCAGTACATAC
AGCTTGCGCTCAAGTTCTTCAGGTGAGGTACCTGGGCCGCCAGAAATAAACACATGCTCAAATTGA
GGCTCTGTGCTTAGCGGATCGGCGCCAATCATCGAATTGTCGGTTGGCAGAACACGATAGCCAATG
ATATCCAAATCAAGGCGTTTCGCATTGCGTTCTAGGATGTCGCGGCATTGTTCGCGTTTGTATTCATC
TTTCGGGAACAAAACGACACCGACACCGTATTTTTCAAAAGAAGGTAGCTTGATACCAAGCTTGACC
GCTTCTTCTAACAAAAATTCGTGAGGCTTTTGCAGCAAAATACCCGCACCGTCGCCACTGCATGGAT
CGCAGCCTTGACCACCACGGTGTTCCATGCGAGCTAGCATATCTAGAGCTTGAGTCACAACCTGAT
GGGATTTGCGGTTTTTGAGATGAGCGACAAAACCGATACCACAGGCATCATGCTCTAGCTCAGGAGT
ATAAAGCCCTTGCGAACTTTGCTCTCTATCTACCATAGATACTTCCTTCCAGTTCTAGAAGGCGCAGC
AACACCAATACGATGAGATCGCATTCTCGGGTTGAGCACCTTGTCCTTTATAGTTTTGAATCGCAATC
AACCAGAGGG

Tag-2:>5107_4465

CGGAATCACGGACTTCCATTGTTCTAACTGAACATGAGCGGCAAGCGCTAGCGAGAGAATCGTTGTA

TCTGGTTTTTTCTCAACCCGCTGAAACCCTTTTGTCGCAACAATGGTCTCACGCCAGTTTTCAACAC
TGTATTGGGCTTGCGCGAGATAACGCCAAATTTCGGCAGCACTCTCATTTTCAGGCACCTGTTTTAG
CAGTTTGTTGAGCTGAGGAATCGCTTTTTTATAATCACCTTGCGTCAGATAGAGTTGCGTCAACATGC
GTCGCGTGACCCACTCTTCTTCAAACGTGAATAATTCGAACGATAGCGCTTTCTCTAACGAGGCAAT
CGAACGCGTGAATTGCTCCGCTTGCCAATAGTAGATCCCCAACAATTTTGACAGCGCTGCCACATCA
TCCGGCCGAGAGACCTCTGCGCTATTGAGCTTCGCGATGGCATCCGCGAGCGCATTATTGTCTGCA
AGCTGTTGTGCATTTTGCATCACGCGTGCGGTGTACTGGCTGACATTGGTACCAGAGGCCGCCAAA
ACAGCGGGTTGCCATAACCATTGAGTGAGCAGTAATGTCAGTATCCGTTTTTTCATCACTGTGCCATC
TTAAATTCTATCTTAGTGGTATAGCCGTAAATGGTTTGAGCTTGTCCATCCACAATCTGTGGCTGGTAG
CGCCAACGCTTAATTGCATCCATCGCATTTCTCTCAAAAAAACGCGCTGGCTGCGCTTCCACCACTT
CAATGTTTTTGGTTCGCCCTGTTTCATCAATATCAAATTTGAGTATGACATAGCCTTCAATACGACGCT
TCTTCGCCTTAGCCGGATACACCGCCTCTACCCGACTTAATGGCATCAACTGTTTGGTGGTATTGCT
GGCGTTTTGCGTCGGCGCACTGACCGTCGCAGGGATAGAAACCGCCAAACCACTGACATTAAAGC
GCATATCAATATTGGGCGTCGCAACCTGTGTCGACGTCACTTGCATCGGCGTTTGCGTCATTTTCGG
CGTTTCCGGTTGTGGCGGCTCAGGGATCTCGGGCGGCGGTGGAACACTACGCTGCCTTCTCGTGA
CTTCGGTATCCTGCTCTACCATCACCATGTTGAAACTCAGTGAAGGCGTCTGATCGATTGCTTGCTTT
TTGCCGCTATTGACGAGCAACGCCATGGCGGAAAACAAGGCAAAGACCAGCGCTAACGCCACGGG
AAACGCGATCACAAGACGCATCATTACGATTTCTCCGCCGCCAATGCGATGTTTTTCACCCCTGCGG
CTTTGGCTGCGTCCATCACTTTAACGACGACGCCGTTGTAGGCGTATTCATCCGCTTGAATCACGAG
CGAAGCATCCGGCTGTTCAAGCAACAGGCGTTCAATGCTCGCTTGTACGCGCTCTACGTCCACCAT
ACGTTTATCGATATAGATGTCATTGGCGGCGGTGATCGCAACGAAAATACCCGCATCTTTCTGCGCC
GAAACGTTGGCCGCTTGAGGGCGATTCACTTCGACACCAGACTCACGCACAAAGGAGCTCGTGAC
AATAAAGAAAATCAGCATGATAAACACAATATCGAGCATTGAAGTTAGGTCAATTTGTGCTTCGTCGCT
TTTACTTTGACGTCTGCCTAATCTCATGAGTCACTCCTCAAAGACTGCTCTAATTTCACTTCTCGGTTT
TGGCACAGTTTTACCAAACGAGCGTGAACAAACATACCTACTAACGCTGCCACCATGCCCGCCATCG
TTGGCAAGGTGGCCAATGAAATGCCAGAAGCCATGAGCTTTGGATTGCTGCTGCCTTGTGTCGCCA
TGACATCAAACACGGAGATCATGCCTGTCACGGTGCCAAGTAAACCAAGCATCGGGCAAATGGCCA
CCAGCACTTTGATAAAGGCCAAGTGTTTGAATAACTGCTGATGCGCGTCATCTAACCACGCTTCTCT
TTGCGAACGAGCAAACCATGAAAGGTGATCGTGTCGCATTTGCCATTTTTGCAATGTTTCTGCACGT
AAACGAGGGTAAGCACAGTAGATAAACAGCAATTTTTCGATGGTCAGTAGCCAAAAGATCACCGCGA
CCGCCGCTAACCACCAGAGCACTTCTCCGCCTTGTTGCATGAAGGAATAGAGCGCATTCCACCATG
AAGATGAAGTCAGCAGTTCTGGCCAAATATCGGAATGAAGGTCCATGATGATCACGCCGCGTTGCC
GACTTTATCCATGGTAGGCGTGAGGCTGGGTAACTGCTGCTCTGCTTGTTGTGCCACCAAACCAATA
CCTTGCTTTTCAAGGATGCTGCGAATTTCTTCCGCCCAACCACTGAGCACGTTATGTGCGAGCAACA
ACGGCATCGCCGCCACCAAGCCTAGCACGGTGGTGACCAAAGCCATCGAGATGCCGCTGGCCATG
ACTTTTGGATCACCGTTACCAAATTGAGTGATCACTTGGAAGGTTTCGATCATACCGATCACCGTACC
AAGCAGCCCCAACATCGGTGCCAAGGCCGCCAACAATTTGAGCATCGAAAGCCCTTTTTCAAGCCC
GGTCTGCTCATCCACTATGGTCTCTAACAAGCGCAGTTCTAGAGCTTCGACACTGCGTGGCTTGTCT
TTGTCATACACCGCAAGCACACGGCCTAGCGGATTATTACCTGGCTGGTTCGGCTGTTTGAGTTGAA
GACGAATTTTGCTCTTGGTGGTCAGCAGCAGAATACCGCGATACAAAGCGATAACGAGCCCCACCG
CAAGCAAGGCAAAGATCACTTTGCCAACCACGCCACCCGCTGCAAAGCGATCGGCAAGGCTTGGC
GAATGCGCGTATTGCTCGAGCAACACCCCGCGAGTGGGATCAATCAACACAGGCACCATTGAAATC
GCGTGCTGTGAACGAAGCGTGTCGAGTGTCGGCGCGCCTTCAGGAAGTTTGGCATAAGGAATCGC
AAAACCTTTGTCGCTCTTCCAGTTAACGAAACCTTGCGGTGCAAGCAAAGCGAAATTCCCCAACGC

CATAACATCAGTGGGTTCAACCGCACCTTCAGCATTGATAAGGTTTACTTGAAGCGCTCGCATTTCAC
CGCTTGCCACAATTTGTTGCTGCATGACATGCCAAAGCGTTTTAAGTTGCGCTATATCGGGCAGCGA
TTGTGCCGCGACAATTTGATTGACCGTTTCAACTTGTTCTGTTTGTCCAATCGCGGTGACTGCGTCG
TTGAGATCGGCTTTGAGTTCTTTCGCGTGCTGGCGAACAACACCAAATAACTCGCCAAGCGAACCC
GTTTCGACACGAAGCTCTTCCTCCAAGTGTGACAAGGTCACTTCGTTATCACTGAACTGACTAGAAA
GTAGCGCGTTTTTCTCTTCCAACGTCGATTTTTGCTTTTCCAGTTGGCGTTTTAATTCGGCCAATTGC
TGCTCTTGGCTGAGAAACGCATTTTCACGCTGTTGGTTTTCTGCCTGTTGTTGGCGGCTTTCTTGCG
TTGCTTTTGGCACTAATTGCGTTTGAGCAAATGATGGGAGGCTCAACGAAACGAGTAAAGCGAGTGA
GATTAATCGACATTTGTTCATTTACTTGGCCTCCGCTGTCGCTAATGCAACGGGCATATTCAACATCG
CGGGCGCGACTTTTTGAGCGGCAATATCAAAGGCACGATTGACATCTGTGCTAGGAATGGTGGTTA
GCGATAACCAGTGACGTTGGTTTTGATCCCAATACCAGAACTGATTGGCATTGAGAGTGCGAGCAAT
AAGCGACACGCGACCAAGATGCAGTTGTTCCACCTCAATCTGCTTACCATCGGCGAGCGTAATAGG
AGCTTGGTAAGCAGACAACTTGCTGCCGTAATCCAACTCAATTTGATACGCCTCTAGAATGCGGCGA
AACTTTTCTGCATCCGCCACATCAGCGCGGACCATCATCTGTTCCAGTTTTTCAATACGCTCTTGAC
GTTGCTGCCATTTGATCGGCATGTCTTGATTGATGGTTGTTTTGAGCGTGTCCAGCATGTCATACATT
AGCGGTACAATGCCCTGACGAGTCACTTGAATGTCTTGAATCTGTTTTGCTAGGCTGCTCTTTTCTT
GTTCTTGATTGGCAATCATGCTGGTCAAGTGACGCTGATAGAGCTCTAGGTTACGAACTTCTTCCTTT
AGGATTTCAATCTCTGCTTGCAGTAACTGACTTTTATCGGCACTCGCATTTATAACGCTTTGGCTCTTA
CTGGCCGCGGTATGAGTACGATTTTGAATTTTCTGTGCTTGTTCAAGGTCGTTACTCATCGCGCCAA
AACTTACGGCCCAAAGAAGTAACGCAGCACCTGCTTTATATCCAGACATAGACGTTTTTGTTACCTAA
AGTGACTACCAAAACAATAAATGATATTTATTCGCATTATCAATGCGACGCATTGTAACGGAATTTTCTC
GCAATACCAAATCGTATGAATAGTAAATCCGTGTTTATTTTGCCATCT

Tag-3:>76_4326
CACCGATCAACGACAGTGGAATGGTGACGATAGGGATGATAACGGCGCGGAACGACCCCAAAAATA
GGGTGATCACCACCAGTACGATCACGGCCGCTTCAACAATGGTTTTCACAACTTCGTGAATGGACTC
ATTGATGGCGATGGTCGAGTCGTACATCACGTTCATCTTGATATTGCTAGGCAGGTTGCGTTCAAGC
TGAGGAAGCAGTTTAAGAACATCTGCCGCAATGTTAATTGGGTTGGCGCTTGGCGCTGCGTTGATG
GCGGCTACCACGGCTTCTTGGCCATTTGCGCTGGCGCGGTAAACATCGTGGCTTTTTTCCAGTGTG
ACTTTTGCGATATCGCCTAAGCGAATCACTTCACCATCACCCGATTTCACCACCAAGTTTTCCAGCTC
TTGTACGTTCGAGACCTGAGTATCGGCACTGCCGTTGTACAGGACAAATTCGCCCGTTACCTGACC
CGTTGCCGATTGGTAGTTGTTGGCGTTGAGTACGCCCATCACGTCGGTTGCAGTGAGGCGTAGTGC
GCCCATTTTGGCAGGATCCAGCCACACGCGTAGTGCGTATTTCAAGCCACCATAGAGGTCAACTTTC
GACACACCATTGATGGTGAAAAGTTGTGGGTTAATCACGCGCTCTAGGTAGTCCGTGATTTGGCTTG
ATGACAGCTCATCACTGGTGAAGCCGATATACAGCACCGCCGTTGTCGAACCGGTCGACATGGTCA
CCGTTGGGTCTTCGGCTTCTTTTGGTAACTGCGATCGAACCGAGTTCGTTTTGGCCAAAATATCCGC
CAAGGCGGCATTCGGATCCGTATTGAGCTTCATGTTCACCGTGATGGTTGATTTACCCAGCACCGAT
TGTGAGGTCATGTAGTCAATATTATCGGCCTGCGCAACGGCTTGCTCCAAAGGTTGAGTAATGAAAC
CTTGGATCAAATCGGCACTGGCGCCGTAGTAACTGGTGGTTACCGTTACGACGGTATTGGTCATTTC
AGGGTATTCTCGCACCTGCATCTTGAACACCGCTTGTAAGCCAAGCAAGGCGATCAAAAAGCTGAT
GGATACCGCTAAAACTGGACGTTTTATAAAAACATCAGTAAAGCGCATGATGCCTCCAATTACAGCAT
AGGGGTTTCAGCCGGAGGTGTAATCGCGTCGCTTTCGACAACACGAACCTTGGCATCGTTACTCAA
GCGAACCTGGCCAGATGTCACCACCTGATCACCCACTTTTACGCCATCGAGAATATGTGCGATATTC
CCAGTGCGTTCACCCACTTTCACCACATGTTGTTTCACACGTTGCACGCCGTCTTCTTCCGTTAGAA

TGTAAACATTGTCACCGTAGAGCGTAAATGTGATCGCCGTTTGTGGCAGTGTGACTTGGTCTTCTAA
CTTCGGCAGAATGATGTTGGCACGGGCAAACATACCACTACGCAGTTTTCCGTCGCTGTTTGGAATA
TCTGCTTGTATTTGAATCAAACCACTTTGGATATTGACCGCGGGTTCAATCGCAGTGATTGAGCCTTT
GAATGGGTTTTGCGGATAAGCATCGACAAAAATATCCACTTCCTGGCCGAGATTGATGCGAGAAATAT
CGTTTTGCGGCACGGTAAAACGTAAGCGCATCACGCTGGTGTCTTCCAAACGTACGATATCAGAACC
TGCTTGTAGGTATTGGCCTAGGTAAACATTACGAATACCAATGACGCCTGAAAATGGCGCTTTGATTT
CACGGCGTGCAATCGAAGCTTTCAAGCTTTCAATGTCGGCTTTTAACGAGTAGTAATTGGCTTCGGC
TTCATCGTACGCTTCTTTTGAAATCGAGCCTTTTTTGAAGAGGCCTTGATAGCGTTTGTACTTCGCTT
CTGCTGCTGGCAGTTTTGCTTCACTGCTCTTCAGGTTGGCTTTTTCTACATCAGAGTCGAGCAAAAC
AAGCGGTTGGCCTTCTTTCACTTGTGTGCCTGATTCAAAGCGGATCTGTTTAATGACACCGCTGGTT
TCATTGGCAATCGTCACACCTTGGTTGGGTTCGATAAAACCGATCGCTTCAATCACAGGCACCCAGT
CGACCGGTTTCACTTCTGTGACCGTTACTGGGAATTCTGGCTCAGGACGATTTGCCAGGTATTCGG
CAATTTTCTTTTGTTTGAAGAGGTTGAAACCTATCACGCTGCCAAACAGCAGAATGGCGATAAGTAAC
ATAAAAAAGGTCCACTTTTTCATTCTATTAAGAACTCCAATTAGTGATTAATGATTGCATCCCAACTGG
CGTCGATGGCAGCGTCAAGTGCCTTATCATCCAGTTGGTAAAAGCCTAATGCGTGTTTTTTCGCTAG
CCTGACGCTGGTCTCCAAACTTAGACCAAACAAAATTTCGTTATCCAGCGTTTTGAACACCCCTTGC
TCCTTGCCCATATCGAACATTAAAGCAACTTGGTGAAACATTTTCTTTTCTAGCTCGCGGGTTTTGTG
GCTATTCGAGCAAGGTAGTGATTCGTACTGAATACGGTTGCTCAAAGTAGACAAGTTTGAAGCCGCT
AAATTCCATGTGTTTAGCCACATGGTGCGATATTGTTGTTTCAAAGGCATATCACGGGTTACGTTGGC
TTGAATCGCCGTGGCAACTCGTTGAGTAATACGTAGCCTGAGTTCATCCAGCAGGTGTTCTTTATCG
TCAAAATAACGATAAATCGTACCTGCTGCGACGCCTGCTTCTTTGGCCAATTTACTCATCGATAGACC
TTGAAAACCAGACTCCGCAATCAACTTCTCTGCCGCAGCCAGGATTTGTTGTTGCTTGTCGTTCTGC
TCTTCAGATGACATGGGTTATCCACCAGTGAATGAACGTTCATTCATTATAGCGGAAAAGATAGCCATA
TTGGCAAGTGCTATGTGTGCTATTGAGTAAAAATCTTGTCAATGAGGCAATGGCAATCTCATCACTCG
ACGACTATTATATGGGCCTCGATTTTTGACCACAGATAAGCGCTATGAAACTGAATCCAAGACAAGAC
GAAGCCGTGAAATACGTATCAGGTCCCTGCCTCGTGCTTGCGGGTGCGGGTTCAGGCAAAACACG
TGTGATCACCAATAAGATTGCCTATTTGGTGCAACAGTGTGGCTACAAAGCGCGTAATATTGCGGCG
GTCACCTTCACCAATAAAGCCGCGCGTGAGATGAAAGAACGTGTGGGGCAAACACTGGGGAAGGG
GGAGTCAAAAGGCCTGATGGTGTCAACCTTCCACACACTTGGTTTGAATATCATTAAGCGAGAGTAT
CGCCATCTGGGTTTGAAGTCGGGCTTTTCCTTGTTTGACGATCAAGATCAGCTTGCACTGCTTAAAG
AGCTGACTGAAAAACAGCTGGATGGCGATAAAGATTTGCTGCGTCAGTTAATGAGCACCATCTCAAA
TTGGAAAAACGACATGCTCTCACCAGAGCAGGCCGTGGCACGTGCGCAAGGCGAACAGCAGCAGT
TGTTTGCCTTCTGTTTTGAGATGTATCAGAAGCAGATGAAAGCGTACAACGCCCTCGACTTTGATGAT
CTTATCTTGCTGCCAGTGTTACTACTTCGTGGGCATGATGAGGTGCGTCACTACTGGCAAAATCGTAT
CCGTTACCTTCTGGTCGATGAGTATCAAGATACCAACACCAGTCAATATGAACTGGTGAAACTGCTG
GTGGGGGAGCGCGGACGCTTAACCGTGGTTGGGGATGATGATCAATCGATTTACTCTTGGCGTGGC
GCGAAACCGCAAAACTTAGTGTTGCTGGGGCAAGATTATCCCAACTTACGCCTGATTAAATTGGAGC
AAAACTACCGCTCGACCAGCCGTATTTTGCGCGCGGCGAATATCTTGATTGCCAATAACCCACACGT
GTATGAAAAGTCGCTCTTCTCGGAAATTCCCGATGGTGAAAAACTCAAAGTGCTGCTGGCCAATAAC
GACGAGCATGAGGCGGAGCGCGTCACGGGGGAAATCATTGCCCACAAGTTTCTCAATCGCACCGA
CTATCGTGACTACGCCATCCTCTATCGTGGTAACCATCAATCGCGTCTCATCGAAAAGTCGCTGATGC
AAAACCGCGTTCCTTATCGACTGTCTGGTGGTACGTCTTTCTTTGCGCGCGCGGAGATCAAAGACAT
CATGGCGTATTTGCGAGTGTTGGTGAATCCTGATGATGACAACGCATTTTTGCGCATCGTCAACACA
CCCAAGCGTGAAATTGGCCCTGTGACGCTGGAGAAGTTAGGCAGCTATGCCAACATGCGTGGCAAA

AGTTTATTTGAAGCCAGCTTTGAACTTGGCCTTGAGCA

Tag-4:>1539_4254 （The tag was used in this study）

GCCATGGTCGAGAAACTGAAATAACGTTTGCAGTTCGTGATAACCATTGTCAGTACGGCCATTGATG
TACAAGAAAAGATTGAGTTTGGCCGGTGAGGGCCAAGTAGTTGGGCTGGTGATCATTATTTTAGCGT
CCACTTACTCACAACGATATTTAATTTGATGTCTTGCTGGGTCAGGCTAAGCTTGGCTGGCAGCGGT
ACAACTTGCTGATGAAATTCCACATCACGATACTGCGTGTAGGCGATATGCCAACGCTGAAAATTGA
GATCTTTCTCTAATGAGCTGAGTGTATTTTGCTCATTGAGTTGAAAGTCGTCAGCGCCATTCGGCAAA
CCGATGATCCAATCACTCAGTTGTTCGACAGGTATAACCAACCCGGTTAACTGTTGGACCAAAGCGG
TCGCATTCGCGGCGGTTAAGATCTGATCGTCGTAGGTTTCAACGCGAGCACCTTGCGGCGTCATGG
TGAGATTTAATGCGGTCTGGCCAAGAAATGTCGTAAAACGCAGGTTGCTTTGCTCTGGTGAGTGTTT
CCAGTAGAAATTGAGGTTTTGTCGTTGATCTGGCGAGATGTAACCCAGTTTGCCAACGGCTTGAAAT
GATTGAATTTGTGACAGTTTGGCTTGATGACTTTGCCACTCAACGCTGGTGGGATGGTCCGGAAGG
CCAGTACAGCCAACCATAAACAGCGGCAAGAGCATTAACCAAAAATAAGTTCGTCGAAACATGGCGG
ATACTTTCTGTCTGATTTAAGTCTTTGAGCGCTCAACTATAGCATCGAACTCACGAAGTAAGGAAAAC
AAATCCGCTACACCCTTTTAATAAAGTCGTGCATAAAGTAAAATTCGCCTCCTAATTTTCCATTCTGTA
CAGAGAAAGCACTATCGATGTCTTTGCTTGCTATTGGCATAAATCACCACACCGCGTCGGTTGACTT
GCGAGAGAAAGTGGCGTTTGGTCCAGACAAACTGGCTAACGCACTGCAGCAGCTCAGCCAGCATG
AAGCAGTCAATGGCAGCGTGATTCTCTCTACCTGTAACCGCACCGAAGTGTATTGCGATGTCAAATC
GGGTGCACGCAGCAAGGTGATTGATTGGTTAAGCCAGTTTCATCAAATCGGTTTAGAAGAGCTCAAA
CCGAGTTTGTATGTGTACGAAGAACAAGCAGCAATTCGCCATCTGATGCGCGTTTCTTGCGGCTTGG
ATTCTTTGGTGCTGGGTGAACCACAAATATTAGGGCAGGTGAAACAGGCGTATTCCGACTCGCGTG
AACAGCAGGCGGTGGATGCGTCTTTGGAAAAATTGTTTCAGAAAACCTTTTCCGTTGCTAAGCGCGT
CCGCACTGAAACCGACATTGGCGGTAACGCCGTTTCGGTCGCGTATGCTGCTTGTACGTTAGCCAA
ACACATTTTTGAATCGTTAGAGAAATCGACGGTTCTATTGGTCGGTGCTGGTGAAACCATCGAGTTG
GTGGCGAAGCACCTTCACGCGAACGGCTGTAGTAAAATGATTGTGGCAAACCGCACTCGCGAGCG
TGCACTGACCTTAGCAGAGCAATTTGATGCTCAGGTGATCAGCTTGCAAGATATTCCCAATCACCTT
GCCAAAGCGGACATAGTGATCAGCTCCACGGCGAGCCCGCTGCCGATCATAGGTAAAGGCATGGT
GGAAACTGCGCTTAAGCAACGTCGTCATCAGCCTATGTTGTTGGTGGATATTGCAGTCCCGCGTGAT
GTGGAAGCGCAAGTGGGCGAATTGAATGATGCTTACTTATATACTGTTGACGATCTGCAATCGATTAT
TGACAACAATATTGAGCAGCGCAAAGTCGAAGCGATCCAAGCAGAAGCGATCGTGGCGGAAGAAAG
TGCCGCGTTTATGAGTTGGCTTCGCTCGCTCCAAGCGGTGGACAGTATTCGTGAGTATCGCCAGTC
GGCCAATGACATTCGTGAGGAATTGCTCAGCAAAGCGCTGTTATCACTGGAAAGCGGTGGTGATCC
AGAAAAAGTACTGCGCGAACTGAGTAATCGCCTGACAAATAAACTCATCCATGCACCCACACGTGCG
CTACAATTGGCCGCAGAGCAGGGTGAGCCCGCAAAATTAACCGTCATTCGCCAAAGCTTAGGGCTG
GATGAACTCAAATAATTCTATCAATCAGTAAGATATATAAATGATGAAAGCCTCGATTCTAACCAAGCTA
GAAATGCTGGTTGAGCGTTATGAAGAAGTGCAGCACCTTCTTGGTGATCCGGGAGTGATCGGTGAC
CAAGACAAATTCCGTGCACTGTCAAAAGAGTATTCTCAACTTGAAGAAGTCACTAAGTGCTTTACTGC
CTATAAACAAGCGCAAGAAGATTTAGTTGCTGCGGAAGAGATGGCCAAAGAAGACGACGCAGAGAT
GCGTGAAATGGCGCAAGAAGAAATCAAAGCGGCAAAAGTGGCCATTGAAGATTTGGCTGCAGAGTT
GCAAATTCTTCTGCTACCGAAAGATCCGAATGACGATCGCAACTGTTTCCTCGAAATTCGTGCGGGC
GCAGGCGGTGATGAAGCCGGTATCTTTGCTGGTGATCTGTTCCGTATGTACAGCAAGTTTGCAGAAA
AACGTGGTTGGCGTATTGAAGTCATGTCCGCGAATGAAGCAGAACATGGCGGCTACAAAGAGATGA
TCGCGAAAGTGAATGGCGATGGCGCTTACGGTTTCTTGAAGTTTGAGTCGGGCGGTCACCGTGTTC

AACGTGTTCCTGCAACCGAATCGCAAGGTCGCGTACACACCTCGGCGTGTACCGTGGCAATCATGC
CTGAAATTCCTGAAGCGGAAATTCCAGAGATCAAAGCAAGCGATCTAAAAATTGATACTTTCCGCTCT
TCTGGTGCGGGCGGTCAGCACGTTAACACCACCGACTCGGCAATCCGTATTACCCACTTACCAACC
GGTACTGTCGTGGAGTGTCAGGACGAGCGTTCACAGCACAAAAACAAAGCCAAGGCGATGGCTGT
TCTAGCGGCTCGTATTGTTCAAGCTGAAGAGGCTAAGCGTGCAGCTGAAGTGTCTGATACGCGTCG
TAACCTACTTGGTTCGGGCGATCGTAGTGACCGTATTCGTACCTACAACTACCCACAAGGCCGTGTG
TCAGATCACCGCATCAACTTGACGATTTACCGTCTTTCTGAAGTGATGGAAGGCGATCTTCAGTCTTT
GATCGATCCTGTGATTCAGGAACACCAAGCGGACCAATTGGCTGCTTTGGCGGAAAACAACTGATC
CCATGTCTGAGTTGATGACACTAGATGCCGCTGTTAAACAGGCGGCATCTCGCTTTTCTGAGTTAGG
CAATGATTCGCCGTCTTTAGACGCCGCGGTATTGCTTTGCCATGTACTGGATAAGCCGCGTAGCTAT
CTGTTTACCTGGCCAGATAAAATACTCACCGAACAAGAGCAGCAGCAGTTTGAAGCCTTAGTGGCG
CGTCGACTGTCTGGCGAACCGGTGGCTTACATCATCGGTGAACGAGAGTTTTGGTCTTTGCCTTTTA
AAGTCGCGCCATCGACGTTAATTCCTCGCCCCGATACCGAGCGTTTAGTGGAGTTGGCGCTAGAAA
AAACTGCGACGCAAACGGGCTCTATCCTCGACTTAGGCACAGGCACGGGAGCGATTGCCATTGCAC
TGGCGTCTGAACTGCCACATCGCACTGTGATGGGCGTGGATTTGCAGCAGGAAGCGAAGCTGCTT
GCTGAGTCGAATGCGTTGGCATTGAATATCAAAAATGTGACCTTTAAACAGGGCAGCTGGTTTGAAC
CTGTGGTTCAAGGCACAAAGTTTGCTTTAATCGTTTCCAATCCTCCTTATATTGATGAGAATGATCCC
CATCTTAATCAAGGAGATGTGCGATTTGAGCCGAAAAGTGCTCTGGTTGCCGAAGAGTCTGGCCTG
GCTGATATCCGTTATATCGCGCAACAGGCACGTGACTATCTTGAGCCTCATGGCTGGCTGATGTTTG
AACATGGCTACGACCAAGGTATCGCTGTTCGAGAAATCCTAGATACGTTGGGATATCAAGAGGTAGC
AACCGAAAAGGATTACGGTGGCAATGATCGAGTGACTTTGGGTTACTTCGTTGCTTTACCAGAAAAC
ACGGATCACTAGATCCAGATATCAAAGAGAACACTATGTACGAAGCTCTGAAATACTTTCATTTACTCA
CCATTGTCATCAGCGCGTTGCTGCTTTCCGTTCGCTATGCATTGATGATG

Tag-5:>50_4225
GAGGTTAAGGGCGTTCAAATACCGTTGCAATGCCTTGGCCTAAACCAATGCACATGGTCGCAAGGC
CGTATTTGGCGTCTTTGGCTTCCATCAGGTTAATCAGAGTGGTTGAAATACGAGCCCCCGAGCAACC
CAGTGGGTGGCCAAGAGCGATCGCGCCACCGTTAAGGTTGACCTTCTCATCCATCACATCCAGCAG
ACCTAGGTCTTTCGCACACGGTAGAGATTGCGCTGCAAACGCTTCATTTAGCTCAATCACATCCATG
TCTTCAATCGATAGACCGGCACGTTTAAGTGCTTTTTGCGTCGCAGGTACAGGGCCGTAACCCATGA
TGGAAGGATCGCAACCGGCGATCGCCATGCCTTTAATACGAGCACGAATCGTCACGCCAAGCTCAT
TGGCTTTCTCTTCACTCATGATCAACATCGCAGACGCACCATCCGACAAAGCAGAGGATGTGCCAG
CAGTGACGGTGCCATTGGCTGGATCAAACACAGGGCGAAGTTGAGAAAGCCCTTCCACTGTGGTTT
CTGGGCGAATCACTTCGTCATGATCCAAGGTGAACAAGGTGCCATCCGCGGCATGCCCTTCGGTGG
GCAGAATTTCGTTTTTGAATCGACCTTCAAGCGTTGCTGCATGGGCACGAGCGTGAGAGCGTGCGG
CAAACTCATCTTGTTGCTCACGGCTAATTCCGTGCAGCTTGCCAAGCATTTCAGCGGTTAAGCCCAT
CATGCCCGCCGCTTTGGCAACGTGTTTCGACATGCCCGGATGAAAATCCACACCGTGGTTCATTGG
CACGTGGCCCATATGCTCAACACCGCCGATCAAACAGATCTCTGCATCACCAGTCATGATGGCGCG
CGCACCATCGTGCAGCGCTTGCATCGATGAGCCACAGAGACGGTTGACCGTCACCGCGCCAATTT
CAATTGGCAAACCTGCCAGCAGCGCTGCGTTTCGTGCTACGTTAAAGCCTTGTTCTAGGGTTTGTTG
AACACATCCCCAATAGATATCTTCAATCTCACTTGGGTTAACTTGTGGGTTACGTGCCAGAATCCCTT
TCATCAAATGGGCTGAAAGATCTTCTGCTCGAGTGTGGCGGAAGGCGCCACCTTTAGAGCGACCCA
TTGGCGTACGTAGACAATCGACAATTACGACATTTTTCATTGTGGGAACTCCTTAGATAGAACCTTGC
TGTTGCGCGCCGTAAAAGGTTTGCCCTCTTTCAGCCATATCAATAAGCATTTGTGGAACCTGATACAT

AGCGCCAAGTTCGACATGCTGCTGTGCCATTGCGACAAAGTTTGCAATACCAACACTATCTAAGTAG
CGGAATACACCACCACGGAAAGGAGGGAAACCAAGGCCATACACCAGCGCCATATCCGCTTCTTGC
GCTGAGGCGATAATGCCTTCTTGCAAGCAAAGCACCACTTCGTTGATCATCGGAATCATCATGCGTT
GGATAATGGTCTGATCGTCAAAGGCTTGTTGAGCAGCACAAACTGGAGCAAGAATCGGTACGATCT
CATCACTGAATGCTTTCTTCGGTTTGCCTTTCTTGTCCATGGTGTACGTGTAGAAACCGCTGCCGTTT
TTCTGGCCATACTTGTTAGCTTCAAATAATGCATCAATCGCATCGCGACCTTGTTTACCCATGCGCTC
AGGGAACCCTTGCGCCATGACGGCTTGAGCGTGGTGTGCGGTATCAATACCGACCACATCCAATAA
GTAAGCAGGGCCCATTGGCCAACCGAACTTACGTTCCATCACTTTATCGATTTGAGTGAAGTCGGCA
CCATCGCGCAGCAACATGCTAAAACCACCAAAGTATGGGAACAGCACGCGGTTAACAAAGAACCCT
GGGCAGTCATTGACCACAATTGGGGATTTGCCCATTTTGGCTGCGTAAGCAACGACACGGTTGATG
GTTTCATCGGAGGTGTGTTCGCCACGAATGATTTCGACCAAAGGCATGCGATGCACAGGGTTGAAG
AAGTGCATACCACAGAAATTCTCTGGTCGTTTGAGTGATTTGGCCAGCAAGTTAATTGGAATGGTCG
AGGTGTTCGAAGTGAGAACGGTCTCTTCGCTCACTTGCTCTTCCACTTCGCTTAATACGGCTGCTTT
CACTTTCGGGTTTTCGACCACGGCTTCTACGATGATATCGGCGTTATCGATGCCCGCGTAGTGAAGG
CTTGGCGTAATAGAAGCGAGAATGCCGGCCATTTTAAAGCCATCAATCTTGCCACGTTCAAGCTGTT
TGTTGAGTAGCTTTGACGCTTCCGTCATGCCCAGATCCAGTGATGCTTGCGCGATGTCTTTCATGAT
CACGGGCACACCTTTGAGAGCAGATTGGTAGGCTATGCCGCCCCCCATAATACCTGCGCCCAGCAC
TGCTGCACGTTGGGTCTCTTTATTGGCGGATTTTGCTGCTTTCTTCGCTATGCCTTTGATGTATTGGT
CATTGAGGAAAAGACCCACCAAGGCCTTGGCTTCTTCTGACTTCGCCAGTTTAACGAAATGTTTTCT
TTCGATGTCTAGCGCTTGATTGCGCGCAAAACGGGCACCTTCTTCAATCGTGACAACGGCGGTCAT
CGGTGCAGGGTAGTGTGGCCCTGCCACTTGAGCTACCAAGCCTTTGGCCATGGTGAAACTCATCAT
CGCTTCGAGTTTGCTTAGCGTTAACGCTGAGGTTTTTTGCTGGCGACGTGCTTTCCAGTCAATTTTT
TCATTGATGGCATCGGTGAGTGTTTGCAGGGCGGATGCGTAGAGGCGATCGGAATCGACGACAGCA
TCAAGCAAACCAATTTTTAGAGCTTCCTCTGCACGGCAAGCTTTGCCTTGGGTGATGATTTCCATGG
CGCTGTCTGCGCCGATCACGCGCGGCAAACGCACACAACCGCCAAAGCCAGGCATAATGCCGAGT
TTGGTTTCAGGAAGACCAATGCTGGTGGTTTTATCACCAATGCGTAAGTCGGTAGCAAGTACACACT
CACATCCACCGCCTAAGGTATGCCCTTTGACAACCGCGACAGTCGGAACGGGCAAATCTTCCAGTT
TATTGAAGATGCTATTGGCAAATTGAAGCCATTGGTCAAGCTCTTCTTCTGGCTTGGCAAACAGTCCT
AAAAACTCAGTGATATCTGCTCCGACAATAAAGGCTTCTTTGTCTGATGTAAGCATTACGCCTTTCAG
ACCGGAGTGAGCGGTGAGCGCATCAAGCGCTTTGTCGAGTGAAGCAAGGGTTGCTAGATCGAGCT
TATTGACGGAATTTTGTGCGCAGAAAAGGATTTCTGCCACTCCATCTTGTACTTCCTTTACCTGTAGG
GTTTCGGCTTGGTAAATCATTGTCTATCTCCATGACATACAATCTTGGATTGTCTGTATACCTAAGGCG
AACATTCGCCGATGTAGGTATAGTGGAAAGTGTTGTATTTATATTCTGGTTAGACCAGTTTGCTTAGTG
TGGACCTCGGATTGTCTAATTTCAATACATTTTTTAAACAAGTGTTTAACATTGTGCGTTAAAGCAGAT
CTTATGCAGTCTGAATAGGGGCATGCTACACTTGCCGCTCTTGTTGATAGTGCGAATGCCATGAACG
AAAATCCATACCTCATCCCAGCCCAAGCGATTCAATTTGAAGAAGAAATCAAAAAGAGTCTCTTTATTA
CTTATCTTGCTCATACCCCGACGATAGAGGCGGCAAAAGCTTTTGTAGAGAGGATTAAAACGCAACA
TGCCGATGCGCGTCATAACTGTTGGGCGTTTGTTGCTGGGCGACCAGAGGATTCAATGAAGTGGGG
TTTTAGTGATGATGGCGAGCCATCCGGTACAGCAGGAAAGCCGATTTTGGCGCAGCTCTCTGGGTC
GGGTGTTGGTGAGATCACCGCAGTCGTAACACGATATTATGGTGGTATTCGCCTTGGCACTGGTGGA
TTAGTGAAAGCGTATGGCGGTGGTGTGCAGCAGGCGCTCAAGCTGCTTCAAACAATTGAGAAAAAA
ATAACCACAAAACTCAGACTGGAGTTAGACTATGCGTTCGTACCAATTGCGCAATCTATAATGCAGCA
ATTCGACGCTGTTGAGGTTGACGCTCAATACAGTGAGAGCGTCGTGCTGGTGGTTGAACTGGAAGT
GAGAAACGTGGAGATGTTTACTCAAACCATGATCAACAAAAGCAGTGCAAAAGCGCTCGTGACGCC

TTATATCGGGAAATAACA

Tag-6:>940_4213
GGGCACGTTCATATCATGCCTGGCTTTACCGCGGGCAATAAGCATGGTGAGCTGGTCACTCTAGGT
CGTAATGGTTCCGATTACTCAGCAGCCGTGTTGGCTGCATGTCTGCGCGCGGATTGTTGTGAGATC
TGGACGGATGTGGATGGGGTGTACAACTGTGACCCTCGTTTAGTCGAAGATGCTCGCTTATTGAAGT
CATTAAGCTATCAAGAGGCCATGGAGCTTTCATACTTTGGTGCATCCGTTTTGCATCCCAAAACCATT
GCGCCAATTGCTCAATTTCATATCCCATGTTTGATTAAGAACAGCTTCAATCCTCAAGGTGCTGGTAC
GCTGATTGGTCAAGATACAGGCGAAGATAATCTAGCGATCAAAGGCATCACTACGCTCAATAACCTG
ACGATGGTCAACGTGTCTGGACCGGGAATGAAAGGCATGGTTGGTATGGCGAGCCGAGTATTTGGT
GCGATGTCGGCCTCTGGCGTGTCAATCGTTCTGATTACTCAGTCTTCTTCTGAGTACAGTATCAGCT
TCTGTATTGAAGCTGCCGACAGACCTTTAGCCGAACAGGCACTCTCAGATGCTTTTGAGTTAGAACT
GAAAGATGGTCTATTGGAGCCCGTCGAGTTCCTGACTAATGTCTCTATCATCACGCTGGTGGGTGAT
GGTATGCGCACTTCCAAAGGTGTCGCATCACAATTCTTTGCTTCTCTTGCGGAAGTGAGTGTCAATG
TTATCGCTATTGCTCAAGGTTCGTCAGAGCGCGCTATTTCAGCGGTGATCCCAGAGGATAAGATCTC
CCAAGCGATCAAAGCGTGTCATGAAAATCTCTTCAACTCGAAACATTACCTTGATGTGTTTGTTGTTG
GTGTCGGTGGTGTTGGTGGTGAGTTGGTGGATCAGATCCAGCGTCAGCAAGCCAAACTGGCAGAC
AAAGGCATTGTCATCCGAGTGTGTGGTTTGGCTAATAGCAAAGGGCTGTTGTTAGACAGTAACGGCT
TACCACTGGAACATTGGCGTGACCGCATGGCCAATGCATCTGAAGCGTTTAGCCTTGCCAGTTTGAT
TGCCACTGTGCAGCGTAACCATATCATCAACCCTGTGCTCGTCGATTGTACTTCTAGCGACGTGATT
GCTAACCAATACGCAGAGTTTTTAGCCGCTGGTTTCCATGTTGTGACGCCAAATAAGAAAGCAAATAC
CGCGAGTATGGCGTATTACCATCAGCTACGCGATGTGGCACGCAGCTCTCGTCGTAAGTTGATGTAT
GAAACCACGGTTGGTGCAGGCTTGCCTGTGATCGAGAACTTGCAAAATCTGATTTCTGCCGGAGAT
GAGTTGGAGCGATTCAGCGGCATTCTCTCTGGTTCACTTTCCTTTATCTTCGGTAAGTTGGATGAAG
GCATGACGTTGAGTGAGGCGACCAATATCGCCAAACAAAATGGCTTTACAGAGCCGGATCCTCGTG
ATGATCTGTCCGGAATGGATGTGGCGCGTAAACTGTTGATCTTGGCGCGTGAAGCGGGCATGGCAT
TAGAGCTGGAAGATGTCGAAGTGGATCAAGCCTTGCCACCAAGTTTTGATGATTCAGGCAGTGTCG
ATGAATTTATGGCTCGACTACCGGAAGCTGATGCGTACTTCAAGCAACTTTCTGCGCAAGCGGCCGA
AGAAGGCAAAGTACTGCGTTATGTCGGTGAAATCATCGATGGTAAGTGCAAAGTCTCGATCGCTGCG
GTAGATGAAAATGATCCGATGTTCAAAATTAAAGATGGTGAAAATGCGTTAGCATTTTACAGCCGTTAC
TATCAGCCGATCCCGTTAGTATTGAGAGGTTACGGTGCCGGTACTCAAGTGACTGCGGCAGGCGTG
TTCTCAGATGTGATGCGTACTTTAGGCTGGAAATTAGGGGTTTAATCGATGAGTTCAAGCGATATGGC
TGTGGTAGTGTACGCTCCCGCTTCTATTGGTAATGTCAGTGTTGGTTTTGATGTATTAGGGGCGGCA
GTGTCGCCCATCGATGGTACTTTGCTTGGCGATCGAGTCATGGTGAAAGCGGGGAATGAGCCGTTT
TCGTTAAAGACGGCGGGTAGTTTTGTCGCAAAGCTGCCAAGCGATCCGAAAGAAAACATTGTTTATG
ATTGCTGGCGAGTGTTTGCTCGCGAACTCGATAAAAAAGGTCTTGAGCTCAAACCGCTTGAGATGA
CATTAGAAAAGAACATGCCGATTGGATCTGGGCTTGGCTCTAGTGCTTGTTCTATTGTTGCCGCGTTA
GATGCCCTCAATCAGTTCCATGCCAACCCACTGGATGAAATGGAATTGCTCGCACTGATGGGTGAAA
TGGAAGGCCAAATCTCCGGTGGAGTGCACTATGACAACGTTGCACCATGCTATTTAGGTGGCTTGC
AATTGATGCTCGAAGAGCTCGGCATTATTAGCCAAGAAGTTCCGTGCTTCGATGATTGGTATTGGGT
GATGGCCTACCCTGGCATTAAGGTGTCGACGGCAGAAGCGCGTGCGATTTTACCTTCTCAATACCG
ACGTCAAGATGTGATCGCACATGGCCGCCATTTGGCCGGCTTTATTCACGCTTGTCACTCAGGGCA
GCCTGAGCTTGCCGCGAAGATGATCAAAGACGTGATCGCAGAGCCTTATCGTGAGAAACTGCTTCC
CGGTTTTGCCGATGCGCGTAAATATGCCGCTTCTGCGGGGGCATTGGCAACGGGCATTTCTGGCAG

TGGGCCGACACTGTTTAGTATTTGCAAAGACCAAGATGTTGCACAGCGTGTGGCACGCTGGTTAGA
ACAAAATTACGTACAAAATGAAGAAGGATTCGTCCACATTTGTCGTTTAGACAAAAAGGGCTCAATTG
TGACAGGAAGTGAGCTATGAAGCTTTACAACATAAAAGAAAATGACGAACAAGTTTCCTTTGGCCAA
GCCGTGCGCCAAGGGTTAGGCCGTAATCAGGGGCTATTTTTCCCAGCAGAATTGCCGAAATTTGAC
GATATCGATGCATTGCTTGCGCAAGATTTTATCTCGCGCAGCACTGCGATTCTTTCCGCGTTAATTGG
TGATGAGCTAGCAGAAGACAAAGTTAACTCAATGGTGGATGCGGCATTCCAGTTCCCAGCACCGATT
AAACAAGTGAAAGAGGGTGTCTACGCGCTTGAACTGTTCCATGGACCGACACTTGCCTTCAAAGAC
TTCGGTGGCCGCTTTATGGCTCAGTCGTTGGCTGCGGTTTCTGATGGTGGAAAAATCACTATTCTAA
CAGCGACATCGGGTGATACAGGAGCCGCGGTTGCACACGCTTTTTACGGTATGGAAGACATCAATG
TGGTCATTCTTTATCCGAAAGGCAAAATCAGCCCATTGCAAGAAAAGCTGTTCTGCACATTAGGCAA
AAACATTCACACGGTAGCGATCAATGGTGACTTTGACGCGTGTCAGGCGCTGGTGAAACAAGCGTT
TGATGATGCAGAACTGCGTGAAGAGATTGGTCTAAACTCTGCAAACTCAATCAACATTAGCCGTTTGA
TGGCGCAAATCTGCTACTACTTTGAAGCAGCGGCGCAAATGAGTAAGCAAGAGCGCGAGAATCTGG
TGGTTTCGGTACCAAGCGGCAACTTTGGTAACCTTACTGCGGGTTTGCTTGCTAAAGCTCTGGGGTT
ACCGATTAAGCGCTTTATCGCTGCAACGAATGCCAATGATACCGTGCCTCGTTACCTAGAAACCGGT
AAATGGGATCCAAAACCGACTGTCGCGACGACCTCAAATGCCATGGACGTGAGTCAGCCTAATAAC
TGGCCACGAATTGAAGAGTTGTGCCGTGTGAAAGAGTGGGGTTTGGAAACTTTGGGCAAAGGTGC
TGTGTCTGATGAGCAAAGTGCGCAATCGGTGAAAGATCTCTATTCACTTGGTTACCTGTGTGAACCA
CATGGTGCGATTGCCTATCGTGTGCTGGATCAGCAGCTACAAGAAGGTGAAACTGGCTTGTTCCTCT
GTACCGCACATCCGGCGAAGTTTAAGGAAGTGGTCGATGACATTCTTGGCAGTGACATCGATTTGC
CAGCACCTCTAGCAAAACATGCCGTGATGGAGCTGCTCTCTGAAGAGTTGGATGCGGATTTTACGG
CTCTGAAAGAAGTATTGCGTCGCGTGCAATAAGCAGGCATCAATCTCAAAGGTGGAGCTTGGCCTC
CACCCTATCG

Tag-7:>4062_4019

CAACGTTTTACGTCGTTCAGGGCTGCGAATTATAAGAGCATTTTTTTGTGATGCAAGTCCTTCGTAGA
AAAAATCCTTAAATTTTTTCCGTTCGATGAAAAAGCGCACAAAGCAGCTAAAAATTCATCGCCCTAGC
GCGTGAAATCAATACTAAATCTTAAAAATGGTTTGGCGATAGAACTTAAGTTCTGCAATAGATTCCCGA
ATATCATCGAGTGCGAGGTGAGTTCCCTGCTTAGAAAAACCTTTCAACACTTCTGGATTCCATCTTCT
GGTCAATTCTTTCAAAGTACTGACGTCGATATAACGGTAGTGAAAATAGCCTTCAAGCTCAGGCATAT
GGCGGTACAAAAAACGACGATCTTGCCCAATGCTGTTACCGCAGATTGGCGATACTCCTTTAGGTAC
CCACTTTTCTAAGAAAGCGATGGTTTGTTCAACCGCCTCTTGCTCAGTAATTTGACTACCACGCACTC
GCTCTACGAGACCACTACCCGTGTGTGTATTGGTACACCACTCATCCATTTTTGCGAGTTCCTCTTCT
GACTGATGAATCGCAAGTACCGGTCCTTCGGCTAAAATGTTTAGCTCGCTATCGGTGACGATAGACG
CTATTTCAATAATTTTATGAACGTCAGGATCCAAGCCTGTCATCTCAAGATCGATCCAAATTAAGTTCT
GATCGCTAAAGGACATAGATGAGTTGCCTATTTGTTTGCTGCTAAAAGAGGTACTATACCTCAAAATAG
TGATAACAAGATACTTCGATAATGTAGCGAAAGTCGTCATACTTAGCTCGTTATCAGCGAACAAATAAA
GTAGATATCGTGGCAAAAAAGAAGAAGTTAACTCAGGGTCAAGTACGTCGTGTACGCGACAACCAAC
AAAAAAGACTGAAGAAACAAGCCGACACCATTCAATGGGATGAAGCAATGTTGGGTGATAGCCGTA
GAGGGCTAGTGATCACACGTTTCGGTCAACACGCAGATATTGAAGATGCCGAAACCGGGCTGATTG
AGCGCTGTAATTTGCGTCGTGGTATTGAATCACTGGTTTCCGGTGACAAGGTCATTTGGCGCAAAGG
CTTAGAGTCAATGGCCGGAATTTCAGGCGTGGTTGAAGCTGTAGAGCCGCGTACTTCAGTGTTAACT
CGACCTGACTATTATGATGGTTTAAAACCCGTTGCGGCCAATATTGATCAAATGGTCATTGTCTCATC
GGTGCTGCCAGAGTTGTCACTCAATATCATTGATCGCTACTTGATTGCGGCAGAGACCCTTGGTATC

GAGCCGCTGTTGGTGTTAAACAAAATCGATTTGTTGCAAGAAGCCGAGCTCGCTACGTATCGTGAGT
GGTTGGCTGACTATGAGAAAATTGGCTACAAGATCCTTTATGTGAGCAAACGCTCTGGCGCTGGCAT
TGCAGAGCTCGAAGCGCAATTGCAAGATCGCATCAATATCTTCGTCGGACAATCAGGCGTGGGTAA
GTCGAGCTTGGTGAATGCGCTCATTCCTGAGCTGGATATCGAAGAAGGGGAAATCTCCGAGCTATC
CGGTTTGGGCCAACATACGACAACAGCAGCACGCCTCTATCACATTCCGTCAGGTGGTAACTTGATC
GACTCTCCAGGGGTTCGTGAGTTTGGGCTCTGGCACTTGGAGCCAGAAGAAATCACCAAAGCCTAT
CTTGAGTTTCGTCCTTACTTGGGCGGCTGCAAGTTCCGAGATTGCAAGCACGCGGATGATCCAGGC
TGTATCATTCGTGAGGCTGTAGAAAATGGTGAGATCAGCGAAGTACGCTACGATAATTACCATCGCAT
TATTGAAAGCATGGCGGAGAACAAAGCCAATCGCCAATATTCGCGCAATAAGAAAGCGGACTTATAA
CGCGTATTGTTGTGTAAGTTTTCGTCACCGATTACTGACAAGTGGAATAATTTTCAGTAACATCACGC
GCCCGAAACAAGAATCAAACAGACATCATCGGTATTTATAATGGATAAGATTAAAGTTGGACTGCAGT
ATTGGATCCCACAACATGGCCTGACTCGCCTAGTGGGTAAATTGGCTTCCGCCAAAGCAGGCAGCC
TGACCACAGCGATCATTCGCTGGTTTATCAAGCAGTACAACGTCAATATGGACGAAGCGAAACACGC
CGATCCTAAACACTACAAGACGTTCAATGAGTTCTTTGTGCGTGAGCTAAAAGAAGGCGCACGTCCA
ATTGCTGAAGGTGATGCGATCATTACCCACCCAGCGGATGCTTGTGTCAGCCAGTTTGGCCCTATTA
CCAACGGCCAATTGATTCAAGCGAAAGGGCATGACTTCTCCGCTCAAGAGCTGTTAGGCGGTGATG
CTGCTCTTGCTGAAGAATTTAAAGACGGTAGCTTCGCGACGTTGTATCTGTCACCTCGTGATTACCA
CCGTGTACACATGCCATGCGATGGTACTCTGCGCCAAATGATTTACGTTCCTGGTGATCTTTTCTCG
GTGAATCCTCTGACAGCAGAGAATGTACCCAACCTATTTGCTCGCAATGAGCGCGTGGTATGTATCT
TTGATACTGAGTTTGGTCCTATGGCTCAAGTGCTTGTTGGTGCGACTATTGTTGGTAGTATTGAACAA
GTATGGGCGGGTACCATTACCCCACCTCGTGGCAATACGGTATACAAATGGGATTATCCAGCTTCTG
GTAACCATGCGGTTATCTTGAAGAAGGGCGAAGAAATGGGTCGCTTCAAACTCGGATCAACCGTCAT
TAACCTTTTTGCCAAAGACGCGATTCGCTTTGATGAGTCGATGGCCAATGGCCAACCAACGGTAATG
GGCACCCCATACGCCCACCAGCAATAAGTTTATTTTTAGCCCCAACAACCGTTGGGGCTTTTTCTTG
ACGAAAAACAAAACTCTTCATAAAAAATCAACGGAATTCAGAAACTTCAACCCGCCTCTAAGATGTTC
TTTGCACAATGAATTAACCTAAGTTTTCACGTGAAATCTTGAGGTGTGATGAATGCAGAAATTGAACA
TGACTGTGGTATGGAAGCTGCTGGTTTGTTTTGCGCTCCCTATCGGCATTTTGTTAATGCCCATCGAT
GCGATTCCTATCGATGATTTGACGCTCATTCAGCATCGACTACTGGCCATCTTTCTACTTGCAGCCTT
ACTTTGGGTTTTAGAGCCCGTGCCCGTTTTTGCCACTTCAATTCTGATCATCGCCCTCGAGCTGATC
ATGATTTCCGATAAAGGATTACATCTGTTCCGTACCCCGCCCGCCGGACACGATCTCGGTGAGTTGA
TTGGTTATACCGACATTTTTTCTGCGTTTTCCTCGCCAATCATCATCCTTTTCATGGGCGGATTTGCAC
TCGCCATTGCCGCATCAAAATACGAGTTAGATAACAACCTCGCTCGGGTACTGCTCAAGCCTTTTGG
CCAGCAACCCAAATTCATTATGTTGGGATTGATGCTGATTACCGCCGTGTTTTCGATGTTTATGTCCA
ATACCGCGACTACGGTCATGATGCTGGCGTTATTGGGGCCCATCGTTGCCTCGGCACCGAAAGGCG
ATTTGGGCATTAAAGCGCTAGTATTGTGCATCCCAATTGCGGCGAACACGGGTGGTATTGCGACCCC
GATTGGTACCCCGCCCAACGCCATTGCGTTGCAATATTTAACTGGAGAAAACAGCATCGACTTTCTC
AGCTGGATGGCAATGGGTTTACCCTTTGTTCTTGTTCAGTTGACCATTGCTTGGCTTTTATTGCAGAA
ACTCTTTCCTTCTAGCGAAGCGAAAATGACGCTGAAATTAGACGGCAAGTTCAAGAAAAGCTGGCGT
GCCATGGTGGTGTATATCACGTTTGCCGCCACGATTTTGCTCTGGATGACCACGCAATTGCACGGCA
TGAATACCTATGTGGTGGCCATCATTCCGCTGGCAGTGTTTACATTGACAGGTATTATGGGCAAAGAA
GAACTCAAGCTGATCAATTGGGACGTACTTTGGCTGGTCGCGGGGGG

Tag-8:>3648_3953

TGGTTGTACTTAGCAACACGGTCAGAACGGCTCATAGAACCAGTCTTGATTTGACCTGCTGCAGTAC

CTACCGCTAGGTCAGCGATAGTTGCATCTTCAGTTTCGCCAGAACGGTGAGAGATTACTGCTGTGTA
ACCTGCGTCTTTAGCCATCTTGATTGCAGCTAGAGTCTCAGTTAGAGAACCGATTTGGTTGAACTTG
ATAAGGATAGAGTTAGCAATGCCTTTCTCGATACCTTCAGCTAGGATCTTAGTGTTTGTAACGAATAGA
TCGTCACCAACTAGTTGGATCTTGTCGCCTAGAAGTTGAGTTTGGTGTGCGAAACCAGCCCAATCA
GACTCGTCTAGACCATCTTCGATAGATACGATTGGGAATTGCTCAACTAGACCAGCTAGGTAGTGGTT
GAACTCTTCAGAAGTGAACGTTTTGCCTTCACCTTTCATGTTGTAGATGCCAGCTTCTTTGTCGAAG
AACTCAGATGCTGCACAGTCCATAGCAAGAGTAACGTCTTTACCTAGTACGTAACCCGCTGCTGCAA
CTGCTTCTGCGATAACTTCTAGCGCTTCAGCGTTAGACTTAAGGTTAGGAGCGAAACCACCTTCGTC
ACCAACAGCAGTGTTGTAGCCTTTAGACTTAAGAACTTTAGCTAGGTTGTGGAATACTTCCGCACCCA
TGCGAACTGCTTCTTTCAGAGTTTTTGCGCCAACTGGTTGGATCATGAACTCTTGGATGTCAACGTT
GTTGTCTGCGTGCTCACCACCGTTGATGATGTTCATCATTGGTAGAGGCATAGAGAACTGACCAGCA
GTACCGTTTAGCTCAGCGATGTGCTCGTATAGAGGCATGCCTTTAGAAGCAGCCGCTGCTTTAGCGT
TAGCTAGAGAAACAGCTAGGATTGCGTTCGCACCAAACTTAGATTTGTTTTCAGTGCCGTCTAGCTC
GATCATGATAGCGTCGATTGCTGCTTGATCTTTCGCATCTTTACCAACTAGAGCGTCAGCGATTGCAC
CGTTTACCGCTTCAATAGCTTTAAGAACACCTTTACCTAGGAAACGTGCTTTGTCACCGTCACGTAGC
TCAAGCGCTTCGCGAGAACCAGTAGATGCGCCAGATGGAGCTGCTGCCATACCTACGAAACCGCCT
TCTAGGTGTACTTCAGCTTCAACAGTTGGGTTACCACGTGAGTCGATGATTTCACGACCTAGAACTTT
AACGATCTTAGACATTAATGTTTCCTCTCGTTCAAATATAAATGTCAAATTTAAAGGGCGGCAGCACAA
CCTTTGCAACCGCCCGTATCCTTTTACTTCAGTTCGCCGCGCGCGTTTTCACCCGCTGCTTTAATAA
AGCCAGAGAACAGTGGGTGACCGTCACGTGGCGTAGAAGTGAACTCTGGGTGGAACTGAGCTGCC
ACAAACCATGGGTGGTTCGGGTTTTCGATCATCTCAACCAGTTTCTTGTCCGCAGACAGACCGGATA
CTTTTAGGCCTGCTTTTTCAATTTGTGGACGAAGATTGTTGTTCACTTCGTAACGGTGGCGGTGACG
TTCATGGATCGTATCGCTACCGTATAGCTCACGAGCTTTAGTGCCTTTCGCTAGGTGACATAGTTGCG
AACCTAGACGCATCGTACCACCAAGATTTGACGATTCAGTACGTTCTTCAACGTTACCTTCACTGTCC
ACCCATTCCGTGATCAAACCTACCACAGGGTACTTGGTGTCTTTGTTAAATTCTGTTGAATGTGCACC
TTCCATACCCGCAACGTTGCGCGCGTATTCAATCAGTGCCACCTGCATACCTAAGCAAATACCTAGGT
AAGGAACCTTGTTCTCACGAGCGTATTTCGCCGCAAGAATCTTGCCTTCGATACCACGGTCGCCAAA
GCCACCAGGTACAAGGATTGCGTCAAGTCCTTGCAGAACTTCAACGCCTTTGGTTTCTACGTCCTG
AGAGTCTACATATTTAATGTTAACGCTCAAGCGATTTTTCAAACCTGCATGTTTAAGCGCTTCGTTTAC
TGATTTATACGCATCAGGTAGTTCGATGTATTTACCCACCATACCGATAGTAACTTCACCAGTTGGGTT
CGCTTCTTCGTAAATCACCTGTTCCCATTCAGACAGATCCGCTTCTGGCGCATTAATGCCAAAACGT
GCACAAACGAGATCATCAAGACCTTGAGATTTGATCAGTTGAGGGATCTTGTAGATAGAGTCTACGT
CCTTCATGGAGATTACCGCTTTCTCAGGAACGTTACAGAATAGCGCAATCTTCTTACGCTCGTTCGCT
GGGATCATGCGATCTGAGCGGCAAACTAGGATATCTGGTTGGATACCGATTGATAGCAGCTCTTTAA
CAGAGTGCTGAGTCGGTTTTGTTTTCACTTCGCCTGCAGCTGCAAGGTAAGGAACAAGTGTTAGGT
GCATGAACATAGCGCGTTCACGGCCGATTTCAACGGCTAGCTGACGAATCGCTTCCATAAATGGTAG
AGATTCGATATCACCAACAGTACCACCCACTTCAACGATCGCAACGTCATGGCCTTGAGAACCATTG
ATCACGCGGTCTTTGATATCGTTTGTGATATGAGGGATAACTTGGATAGTTGCACCTAGGTAGTCACC
GCGACGCTCTTTCGCAAGAACGTCTGAGTAAACACGACCTGCAGTGAAGTTGTTGCGCTTAGTCAT
CTTAGTGCGGATGAAACGCTCGTAGTGACCAAGGTCTAGGTCTGTTTCGGCGCCATCTTCTGTAAC
GAACACTTCACCATGTTGAGTTGGGCTCATTGTGCCTGGATCAACGTTGATGTAAGGGTCAAGCTTC
ATCATAGTCACTTTAAGACCACGAGCTTCTAAAATCGCTGCAAGAGATGCTGCTGCAATACCTTTACC
TAGAGAGGATACAACCCCGCCAGTAACAAAAATGTAATTTGTCGTCATGTTTAACCTGAAATTGGTTG
AATGAGGGAAACGGATTTCTTCTGGACGGGACGAAAATATACCAGAAGACCCTAACCGCCACAACG

TGAAATCTATCACACGCCAATTTTTTATTTTTTGCTTCAAATCAAAGTGGATAAACACCAATCGAGTTG
AATAAAAAAATAGTTGAGTCAAATGTGTCTCAAATTTCTACTCGGCAGCTTGACTGCGTTCCGCGCGCT
TCACTTGATCCCAAATGGCATCCAACTCTGCTAAATCGCACTCTTCAAGCTTCTTACCTTTTGCGTGT
ACACCTTGCTCTACTTGTTTGAATCGATTAGAAAACTTGAGATTAGCCTTGCTCAATGCCGCTTCCGG
ATTGACCTGCAAATGGCGCGCTAAGTTGACGGTGGCGAACAGCAGATCCCCCAGCTCAAGCTCCA
CTTTTTCCTCATCGACATCGACTTGAAGCGCCTCTTCCACCACTTCCTCAATTTCTTCTTTGACTTTG
TCGACCACAGGCCCGAGTGAGTCCCAATCAAAACCATAATGAGCGCATTTTTTCTGGATCTTTGTAG
CACGCATTAACGCAGGTAGAGAGTTTGGTATTGAGTCTAGAATACTTTGCTCATTTTTGCCTATTTGTG
CTTTCTCTTTGGCCTTTTCTGTTTCCCAATTGGCTTTTATTTCTGCTTCTGTCTCAAATTCGACTTGGC
CGAACACATGGGGATGACGGCGTGTCAGTTTTTCGTTCACGGTTTCTACCACGGAGGAAAAATCAA
ACAACCCCTGCTCTTTTGCTAATTGGCTGTAGAAAATCACCTGGAACAACAGATCGCCCAACTCTTC
CTGCAAATTAGGCCAATCGCGATTATGAATCGCATCCACCACTTCGTACGTTTCTTCGATAGTGTGAG
GAACAATGGTGTCGAAGCTCTGCTTTAAATCCCAAGGACAGCCACTGTGCGGGTCACGCAGCTTCG
CCATGATTTGTTCTAATTGTTCAATTGGATGACTCATTTCTTGTCCCT

Tag-9:>81_3631
TTGCTGGCCGTTAAACTTTAATTGTTGCTTTTTTGTGTGTTTTTTGCCAAATTGTGTTCCGACCTGATT
TTTAGAGTAATATTCTGCAATCAGAAGGGATTCATTGGTTTGTTGAATGATCATCGTGTTTGATTATTCA
GCACCACAGACAAGGCAGTAGAGGTCTGAAATGTCACTATTAGAAGTGAAAAATCTTCGTATCGAGT
ATCCATCGCGGCATGGTATTCATGCGGCGGTGAAATCGTTGTCGTTTAATATTGAACGAGGCGAAATT
GTTGGCGTCGTCGGTGAGTCGGGTGCGGGCAAGTCAACCGTAGGTAATGCGGTGAGCGATTTGTT
GAGCCCTCCTGGTCGTATTGCCAGCGGTGATGTGTTCCTTGATGGCGAAAAAATTTCAGGCCTTTCT
CCAGAAGCAATGCGCAAAGTGCGTGGCTCTAAAATTGGTTTTATCTTCCAAGATCCAATGACCTCGC
TCAATCCTCTCTTTACTGTTGAACAGCAGTTAAAAGAAACCATTCACGCCAATATGAATGTCTCTGAC
GAAGAGGCTTACACGCGCGCATTGTCATTAATGAAGCAGGTGGGCATCCCTCAACCTGAAAACCGC
TTAAAGCAGTATCCTCACCAGTTTTCTGGCGGTATGCGTCAGCGCGTGGTGATTGCGATTGCCCTAG
CAGGCGAGCCGGATTTGATCATTGCCGATGAACCCACCACGGCACTCGATGTGTCGATTCAAGACC
AAATCCTCAACCTGATCCGCGAGCTATGCATTAAGCACAACGTGGGTTGCATGTTGGTCACGCACGA
CATGGGCGTGGTATCGAACGTGACTGATCGTGTTGCTGTTATGTACCGAGGTGACTTGGTTGAATTT
GGCCCAACGGCCAAAGTGTTGGGCAACCCAGATCATCCTTATACGCGCAGCTTGATTTCAGCGGTG
CCGCGTTCGGATAGGAAGCTTGATCGCTTCCCTCTGGTCAGTTACATCGAAGAAGCGAGCGAATTG
CAGCCTCTTGATATTAAGAATCACTGGCTGGGTCAAAGTGAAGATCAGAGAAAATACACCGGGCCGC
TGCTTAATGTGGAAAACGTTAACTTGCGTTTCGTCACTAAGGACTCGCTGTTTGAGAGCCGTCGTGA
GTATGTGCAGGCATCAAATAACGTTAGCTTCCAAGTGCATGAAGGGGAAACCTTTGGCTTGGTGGG
GGAATCGGGTTCGGGTAAATCCACTATTGCACGAGTCATTGCCGGGCTGTATGCGCCTAACTCAGGT
AAGGTGACGTTTGAAGGTATTGACCTTACCGCATTGACCTCAGAAAAAGCGCGCCGTCCTTTGCGT
CGCCAAATGCAGATGGTCTTCCAAAATCCATACACTTCGATGAACCCGCGCATGAAGATATTCGACAT
CATCGCGGAGCCCATTCGTTTCCATAAGTTGACCAACAGTGAAGCGGAAACGCGCCAAATCGTGAA
CGATCTCTTGGAGCACGTTGGCTTAGGGCGAATGGCCGGGGTGAAATATCCACATGAATTTTCGGG
TGGTCAGCGTCAGCGTATTTCGATTGCGCGCGCATTAGCGACTCGTCCACGTCTTTTAATTTGTGAT
GAGCCAACCTCGGCACTGGATGTCTCGGTTCAAGCACAGATTCTGAATTTGCTCAAAGATCTACAAA
GCGAACTGAACTTAACCATGCTGTTTATCAGTCATGACCTTCCGGTTATCCGCCAAATGTGTGACCG
AGTCGGTGTGATGCAGATGGGAACCCTGCTCGAAGTCGCACCGACTGAGCAACTGTTCCGCTCTC
CACAGCATGAATACAGTAAGCACCTGATTTCCTTGATGCCAGAGTTCACTGGTTTGAGGGAAGAGGT

GAAAACGGCATAACCAAGCTTGCTTGGCACAATAACAGACGCAAATACAACAACTAGGGATTCGGAT
TCCCGCATAAGGAGTTATGCAATGAAAACCATGAAAAGCAAACTGACCGTGGCTTTAATGGCTGCAG
GCCTAAGCCTGAGTGCTGCTGCGGCAGACATCAAAGTTGCTTATGATGCAGATCCAGTGTCACTTG
ACCCGCATGAGCAGTTGTCTGGTGGTACGCTACAAATGTCGCACATGGTGTTTGATCCACTAGTTCG
TTATACCCAGAAATTGGATTTTGAACCTCGCTTGGCTGAAAAATGGGAACGTGTCAATGACACCACTT
ACCGCTTCCAGCTACGTAAAGGGGTGAAGTTCCATTCCGGTAACGAACTGACGGCAGATGATGTCG
TGTGGACATTCGATCGTCTGAAAGATTCTCCAGACTTTAAAGCGATCTTTGAACCGTATGAAAAAATG
GTCAAAGTGGACGATTACACCGTTGAGTTGGTGTCCAAAGGGGCTTACCCATTGGTGCTACAAACC
GCCACTTACATCTTCCCGATGGACAGCAAGTTCTACTCTGGCACGACAGCCGATGGCAAAGACAAA
GCGGAAGTGGTGAAGCACGGTAACTCATTTGCTTCGACCAACGTTTCTGGTACGGGTCCGTTCATC
GTCACTTCTCGTGAGCAAGGCGTGAAAGTAGAGTTTGAGCGCTTTAAAGATTACTGGGACAAAGAG
TCAAAAGGTAACGTTGATAAGCTGACGCTTGTGCCAATCAAAGAAGACGCGACTCGTGTCGCTGCG
CTCCTTTCTGGTGGTGTTGACATGATTGCGCCAGTGGCACCTAACGATCACCAACGCGTGAAAGAT
GCGAAGGGCATCGATCTTGTGACGCTGCCTGGTACGCGTATCATCACTTTCCAAATGAACCAAAACA
GCAACGAAGCGCTGAAAGATGTGCGCGTGCGTCAGGCGATCGTTCATGCCATCAACAATCAAGGTA
TCGTTGATAAGATCATGAAAGGTTTCGCGACAGCGGCTGGCCAGCAGGGCCCTGAAGGCTACGCG
GGCTACAACGCGGAGCTGGTTCCTCGTTTCGATCTGAAAAAAGCGAAGCAGTTGATGAAAGAAGCG
GGTTATGAGAAAGGCTTTACGCTGACGATGATCGCGCCGAACAACCGTTACGTTAACGATGCGAAA
GTGGCTCAAGCGGCGTCTGCCATGCTATCGAAAATCGGTATCAAGGTAGACCTAAAGACCATGCCTA
AAGCGCAATACTGGCCTGAGTTTGATAAGTGTGCAGCAGACATGTTGATGATCGGCTGGCACTCAG
ACACAGAAGATTCGGCGAACTTCTCTGAGTTCCTCACCATGACGCGTAACGAAGAAACGGGCCGTG
GTCAGTACAACTGTGGTCACTACTCAAACCCAGAAGTGGACAAGTTGGTGGAAGCGGCGAACGTAG
AAACCGATCCTGCTAAACGTGCAGCGATGCTACAGAAAGTCGAAACCACGCTGTACAACGAAGCAG
CATTCGTTCCTCTACACTGGCAAAACCTAGCGTGGGGCGCGAAATCTACGCTGGATATCAAACCAAT
TGTCAACGCGATGGACTTCCCATACTTTGGTGACTTGGTAGTTAAAGAGTAACACTCGCTTTCTCCC
ACTCAAGCGTTGGGTGGGATGAGTGATTTTAGGCGCTCAACTTTGTTTCAGTCGGGTTGAGCGCCG
TTTTCAGACTGAAGTTTATATGGCCGTGATAGC
